# The epigenetic regulator SETDB1 as a key component of cancer stem cells and drug resistance in primary liver cancer

**DOI:** 10.1007/s13402-025-01157-3

**Published:** 2026-01-06

**Authors:** Maël Padelli, Christophe Desterke, Aurore Devocelle, Georges Uzan, Antoinette Lemoine, Julien Giron-Michel

**Affiliations:** 1 INSERM UMR-S-MD 1197, Ministère des Armées et Université Paris Saclay, Villejuif, France; 2 Department of Biochemistry and Oncogenetics, Paul Brousse Hospital, AP-HP, Villejuif, France; 3 INSERM UMR1310 et Université Paris-Saclay, Villejuif, France; 4 INSERM UMR1193 et Université Paris-Saclay, Villejuif, France

**Keywords:** SETDB1, Hepatocellular carcinoma, Cancer stem cells, Drug resistance, Epigenetics, Paclitaxel, Immunotherapy, Liver cancer

## Abstract

**Background:**

Hepatocellular carcinoma (HCC) is a highly heterogeneous malignancy with a poor prognosis and limited response to current therapies. Cancer stem cells (CSCs) contribute to this heterogeneity, driving tumor progression, immune evasion, and therapeutic resistance. Epigenetic regulators have emerged as pivotal modulators of CSC phenotypes. The histone methyltransferase SETDB1, a key stemness factor in both embryonic and adult stem cells, catalyzes H3K9 trimethylation (H3K9me3) and acts as a major oncogenic driver in a wide range of solid and hematological malignancies. Yet, its specific role in maintaining stemness and contributing to therapeutic resistance in HCC remains poorly defined.

**Methods:**

Integrative analyses of bulk and single-cell transcriptomic datasets from public HCC cohorts were performed. Tumors were stratified by SETDB1 expression, followed by pathway enrichment, immune deconvolution, and stemness scoring. Associations with clinical outcomes, immune phenotypes, drug resistance signatures, and chromatin-binding partners (DNMT3A, TRIM28, HDAC1) were also explored. The role of SETDB1 was then examined in vitro, where a stem-like phenotype was induced in HCC cell lines under hypoxic conditions, and patterns of SETDB1 expression and stemness marker levels were assessed following paclitaxel treatment.

**Results:**

SETDB1-low tumors were enriched in well-differentiated, immunologically active subclasses and were associated with increased predicted responsiveness to immune checkpoint blockade (ICB), sorafenib, and transarterial chemoembolization. Conversely, SETDB1-high tumors exhibited transcriptional features of dedifferentiation, elevated stemness scores, and enrichment in aggressive molecular subtypes. These tumors also displayed higher TP53 mutation rates, reduced immune infiltration, immune exclusion signatures, and activation of pathways linked to ferroptosis and ABC transporter dysfunction, consistent with resistance to ICB and conventional therapies. SETDB1 expression was also associated with hypoxia-related pathways, suggesting a role in maintaining CSC niches. In vitro, hypoxia-induced stemness coincided with increased SETDB1 levels, whereas paclitaxel treatment decreased both SETDB1 expression and stemness markers. A chromatin risk score incorporating SETDB1 and its partners predicted poor disease-free survival independently of clinical parameters.

**Conclusions:**

SETDB1 defines a stemness-enriched, immune-resistant HCC subtype associated with poor outcomes and therapeutic failure. Correlations between SETDB1 expression and stem-like features in vitro suggest a potential role in maintaining CSC phenotypes, supporting its relevance as a biomarker and candidate for epigenetic- and CSC-directed therapeutic strategies.

**Supplementary Information:**

The online version contains supplementary material available at 10.1007/s13402-025-01157-3.

## Introduction

Primary liver cancer, predominantly hepatocellular carcinoma (HCC), is the third leading cause of cancer-related mortality [1]. Despite recent advancements in traditional treatments such as surgical resection, transarterial chemoembolization (TACE), chemotherapy, and immunotherapy, the 5-year survival rate for HCC remains dismal, often under 20%, largely due to drug resistance and disease recurrence [2].

A defining feature of HCC is its remarkable heterogeneity, both across patients (interpatient) and within individual tumors (intratumoral). Interpatient heterogeneity plays a central role in the stratification of patients for personalized medicine. However, intratumoral heterogeneity (ITH), which refers to the coexistence of genetically, epigenetically, and phenotypically distinct subclones within the same tumor, poses an even greater challenge to treatment success. ITH has been recognized as a major contributor to tumor progression, immune evasion, therapy resistance, and ultimately, poor clinical outcomes [3]. The most aggressive cancer cells display characteristics similar to progenitor cells, leading to their designation as cancer stem cells (CSCs). They exhibit significant self-renewal capacity, the ability to form metastases, and the potential to differentiate into various cancer cell lineages [4, 5], all of which grants them high tumorigenicity [6]. In HCC, CSCs have been identified using surface markers such as CD133, CD44, EPCAM, and CD24 [7]. CSCs are thought to be responsible for driving therapeutic resistance, especially to conventional treatments such as chemotherapy, targeted therapy, and immune checkpoint blockade. Moreover, CSCs often reside in protective niches shaped by the tumor microenvironment (TME), where they are shielded from immune attack and supported in their plasticity by hypoxia, stromal signals, and epigenetic modulation [8]. Given their role in cancer’s persistence, targeting CSCs represents a highly promising, novel therapeutic approach for developing more effective and lasting treatments that can lead to long-term remission. However, no therapeutic strategy specifically employing this approach is currently used in the clinic, largely due to a limited understanding of the precise molecular targets that would effectively reduce their stem-like properties.

Alterations in different histone lysine methylations have been associated with tumorigenesis [9] and drug resistance [10]. Furthermore, blocking the enzymes responsible for these modifications, known as histone lysine methyltransferases (KMTs), can even reverse drug resistance [4]. However, the link between KMTs and CSCs remain largely unknown. The histone KMT SETDB1, a key stemness factor both in embryonic and adult stem cells [11, 12], has emerged as a predominant oncogene in a wide range of solid cancers and hematological malignancies [13–15] promoting chemoresistance [10]. Recent analyses in HCC reveal that SETDB1 is overexpressed and strongly associated with advanced disease features, such as tumor microsatellite formation and metastasis. Conversely, its expression inversely correlates with immune cell infiltration [16, 17].

In this study, the expression patterns, regulatory mechanisms, and functional implications of SETDB1 in HCC were investigated using a combination of in vitro assays, in silico analyses, and public transcriptomic datasets (TCGA, GEO). The results showed that SETDB1 overexpression defines a high-risk HCC subtype characterized by stemness enrichment, aggressive behavior, and therapy resistance. These data position SETDB1 as a potential key component of CSCs, a critical biomarker for prognosis, and a promising candidate for epigenetic therapy targeting CSC-driven and immune-resistant liver tumors.

## Material & methods

### In vitro experiment

#### Human cell line culture and hypoxic treatment

Huh7 and HepG2 cell lines from ATCC were cultured in Dulbecco’s modified Eagle’s medium (DMEM; Gibco, France) supplemented with 10% fetal bovine serum (FBS; Gibco, France) in a humidified incubator with 5% CO₂ and ambient oxygen (~ 21%) at 37 °C. For hypoxic treatment, cells were placed in a hypoxia workstation (Baker Ruskinn SCI-tive, Sanford, Maine, USA) and subjected to a gas mixture of 1% O₂, 5% CO₂, with the balance consisting of N₂, for durations ranging from 3 to 48 h.

#### RNA extraction and real-time quantitative RT-PCR

Total RNA was extracted using RNeasy^®^ Mini Kit (Cat. No. 74104, Qiagen, Germany) according to the manufacturer’s instructions. Complementary DNA (cDNA) was synthesized using SuperScript^®^ III System (Cat. 18080044, ThermoFisher, Waltham, MA, USA). Real-time quantitative PCR was then performed using TaqMan Universal PCR Master Mix (Cat. 4318157, ThermoFisher) with TaqMan Gene Expression Assays. Each reaction consisted of 500 ng of starting cDNA and was run for 40 cycles. 18 S was used as the endogenous control for normalization. Relative gene expression was quantified by the 2^−ΔΔCT^​ method. The primers and probes used in this study are listed in Table S1. To ensure the reliability of the results, all experiments were performed in at least triplicate.

#### Flow cytometry

Suspensions of enzymatically detached cells were prepared in staining buffer (0.5% BSA in PBS). 10^5^ cells were stained with conjugated antibodies directed against the cell markers CD24, EPCAM, CD44, and CD133. After rinsing in phosphate-buffered saline (PBS), 10,000 events were analyzed on an LSR Fortessa flow cytometer (BD Biosciences, Oxford, UK). Data were subsequently analyzed using FACS Diva software (BD Biosciences). Three technical replicates were used for each condition, and each experiment was performed at least three independent times.

#### Immunofluorescence staining

The intracellular localization of SETDB1 was examined in Huh7 cell line. For immunofluorescence staining, fixed Huh7 cells were permeabilized with 0.3% Triton-X-100 and then blocked with 0.1% Triton-X-100 + 1% BSA. Cells were incubated with anti-SETDB1 (MA5-15722, ThermoFisher, Plainville, MA, USA) overnight at 4 °C. After incubation with a fluorophore-conjugated secondary antibody (A-11001, ThermoFisher, Plainville, MA, USA), the glass coverslips were counterstained with DAPI and observed under a fluorescence microscope.

#### Western blotting

Protein extraction was performed using RIPA buffer (catalog P00013C, Beyotime, Shanghai, China) supplemented with cOmplete protease inhibitor cocktail (cat.4693116001, MilliporeSigma, Burlington, MA, USA) according to the manufacturer’s instructions. Protein concentration was determined using the Pierce BCA Protein Assay kit (catalog 23225, Thermo Fisher Scientific, Waltham, MA, USA). A total of 20 µg of protein per sample was loaded onto an SDS-PAGE gel and subsequently transferred to a PVDF membrane. Immunodetection was performed with the following primary antibodies: SETDB1 (ab-107225, Abcam, 1:750), HIF1A (cat.610359, BD Biosciences, 1:1000), OCT4 (AF1759, Biotechne, 1/1000), NANOG (sc-134218, SantaCruz Biotechnology, 1/200), CD133 (130-092-395, Miltenyi Biotec, 1/200), and β-actin (sc-47778, SantaCruz Biotechnology, 1:250), which was used as a loading control.

#### Cell viability assay

Response to sorafenib chemotherapy was assessed by the CellTiter-Glo luminescent cell viability assay (Promega Corporation, Madison, WI, USA). Cells were plated at 3000 per well in 96-well microtiter plates and incubated for 48 h at 37 °C under normoxic or hypoxic conditions. Subsequently, increasing concentrations of sorafenib (0 to 10 µM) were added to the wells, and the cultures were incubated for an additional 48 h. Cell viability was determined using the CellTiter-Glo luminescent cell viability kit from Promega Corporation (Madison, WI, USA) according to the manufacturer’s instructions. The half-maximal inhibitory concentration (IC50), was calculated by nonlinear regression analysis using GraphPad Prism software (San Diego, CA, USA). The experiment was performed with at least three independent replicates.

#### Statistical analysis

GraphPad Prism (v.5.0) was used for statistical analysis, and *p* < 0.05 was considered statistically significant. Wilcoxon rank sum test were employed in two-group comparisons to evaluate differentially expressed genes or microRNA (miRNAs).

### Public transcriptome data collection and preprocessing

#### Bulk RNA-seq transcriptome analyses

Bulk RNA-sequencing data for liver hepatocellular carcinoma (LIHC) were retrieved from The Cancer Genome Atlas (TCGA) program [18]. Both normalized mRNA expression profiles (RNA-Seq by Expectation Maximization, RSEM), somatic mutation data, microRNA expression data, and comprehensive clinical annotations were obtained via the Genomic Data Commons (GDC) Firehose pipeline through cBioPortal (https://www.cbioportal.org/, accessed February 13, 2023) [19] or the LinkedOmics platform (http://www.linkedomics.org/*)* [20]. For analyses requiring raw count data, RNA-seq count matrices were normalized with edgeR Bioconductor R-package version 3.38.4 to account for library size and composition bias. Clinical data were integrated with expression matrices using the *dplyr* R package (v1.0.10). Only primary liver tumor samples with complete transcriptomic and clinical information were retained, yielding a final cohort of 364 patients for downstream analyses. Patients were stratified into two groups (SETDB1 high (SH) and SETDB1 low (SL)) based on the median SETDB1 expression (*n* = 182 per group) (Table S2). Differential expression analyses compared transcriptomic profiles between SH and SL tumors. Functional enrichment analyses were performed to identify pathways and gene sets associated with SETDB1 expression. Molecular and cellular phenotypes were computed using Gene Set Enrichment Analysis (GSEA) and single sample gene set enrichment analysis (ssGSEA) with predefined signatures from the Molecular Signatures Database (MSigDB), including KEGG (Kyoto Encyclopedia of Genes and Genomes), WikiPathways, Hallmark, and published HCC gene sets (Table S3). Enrichment scores for each tumor sample were generated with GenePattern (www.genepattern.org/, accessed on 30 April 2020). ssGSEA scores reflect coordinated up- or downregulation of gene sets within each sample [21], based on the Matthews correlation coefficient (MCC) for robust performance across all prediction categories [22]. In GSEA, significance was set at false discovery rate (FDR) < 0.25 and *p* < 0.05, and the most enriched pathways were selected based on normalized enrichment score (NES). The GSE104580 cohort was used as an independent validation dataset, in which patients were stratified into two groups based on SETDB1 expression levels (SH, *n* = 74; SL, *n* = 73).

#### Web tools used to analyse SETDB1 expression and correlation with stemness and immune microenvironment

SETDB1 mRNA expression in TCGA-LIHC tumors versus adjacent non-tumorous tissues was assessed using the University of Alabama at Birmingham CANcer data analysis Portal (UALCAN, http://ualcan.path.uab.edu/, accessed November 9, 2023). SETDB1 protein expression was further assessed in the Clinical Proteomic Tumor Analysis Consortium (CPTAC)-LIHC cohort via UALCAN. Group comparisons were performed using Wilcoxon rank-sum tests. The correlation between SETDB1 expression and the most frequent driver mutations reported in LIHC [23] was examined using the Tumor Immune Estimation Resource (TIMER 2.0) databases (http://timer.cistrome.org/, accessed November 9, 2023), with correlations evaluated by Spearman’s test. SETDB1 expression in TACE and sorafenib responders versus non-responders was examined using HCC transcriptomic datasets GSE104580 and GSE109211 from the Gene Expression Omnibus (GEO) database (https://www.ncbi.nlm.nih.gov/, accessed on 7 November 2023) using the GEO2R tool. Stemness features were quantified using CancerStemnessOnline (http://bio-bigdata.hrbmu.edu.cn/CancerStemnessOnline, accessed on 7 November 2023), focusing on the mRNAsi (mRNA-based stemness index) and Stemness Index, where higher values indicate a more progenitor-like phenotype. Immune infiltration was estimated with xCell (https://xcell.ucsf.edu/, accessed on 7 November 2023), and differences in immune cell distributions between SETDB1 groups were compared. Correlations between SETDB1 and gene expression or stemness scores were assessed by Spearman’s test, with *p* < 0.05 considered significant.

#### Single cell transcriptome analyses

Single-cell transcriptome analysis was performed using the HCC dataset GSE125449, which was downloaded from the NCBI website [24]. Bioinformatic analyses were conducted using the R software environment (version 4.0.2) and the Seurat R-package (version 3.2.2). In the preprocessing step, genes with positive expression in fewer than three cells were removed. Following canonical correlation-based batch correction, the merged dataset was normalized and scaled. Dimensionality reduction was performed using principal component analysis (PCA), followed by t-SNE for data visualization. The single-cell stemness score was calculated in Seurat by integrating the expression levels of the markers GDF15, ALDH1L1, GPC3, AFP, EPCAM, CD44, and CD24. Data visualization was generated using the FeaturePlot, DotPlot, and VlnPlot functions from the Seurat R package.

#### Prognosis risk score determination

Protein-protein interactions (PPI) for SETDB1 were determined using the STRING network server (version 11.5; confidence threshold > 0.7; Homo sapiens) on February 11th, 2023. A functional enrichment analysis on the resulting PPI network was performed using the Gene Ontology Molecular Function database.

For the TCGA liver cancer cohort, a SETDB1-related expression risk score was computed from partners with a univariate Cox p-value < 0.05 and an unfavorable expression profile. This score was calculated by summing the products of the Cox beta coefficients and the RNA-seq expression levels of these markers. The risk score threshold was determined using standardized log-rank statistics with the survminer R-package (version 0.4.9) and a loop of univariate Cox survival analysis was performed with the loopcolcox R-package (version 1.0.0).

A multivariable Cox model for disease-free survival (DFS) was built with the survival R-package (version 3.5-0) to include the SETDB1-related risk score. The model also integrated demographic parameters (age, gender, weight), liver cancer risk factors (hepatitis B, hepatitis C, NAFLD, alcohol consumption), and pathological classification (grades 1–4). The model was calibrated by bootstrapping with the rms R-package (version 6.4-1), and a corresponding nomogram was drawn with the regplot R-package (version 1.1).

Finally, expression analysis on TCGA RNA-sequencing data was performed via PCA with the prcomp R-base function. Expression heatmaps were generated with the pheatmap R-package (version 1.0.12), and unsupervised clustering was performed using Euclidean distances and the Ward.D2 method.

## Results

### SETDB1 expression is upregulated in HCC and correlates with genomic alterations, aggressive tumor features and poor prognosis

**Fig. 1:**
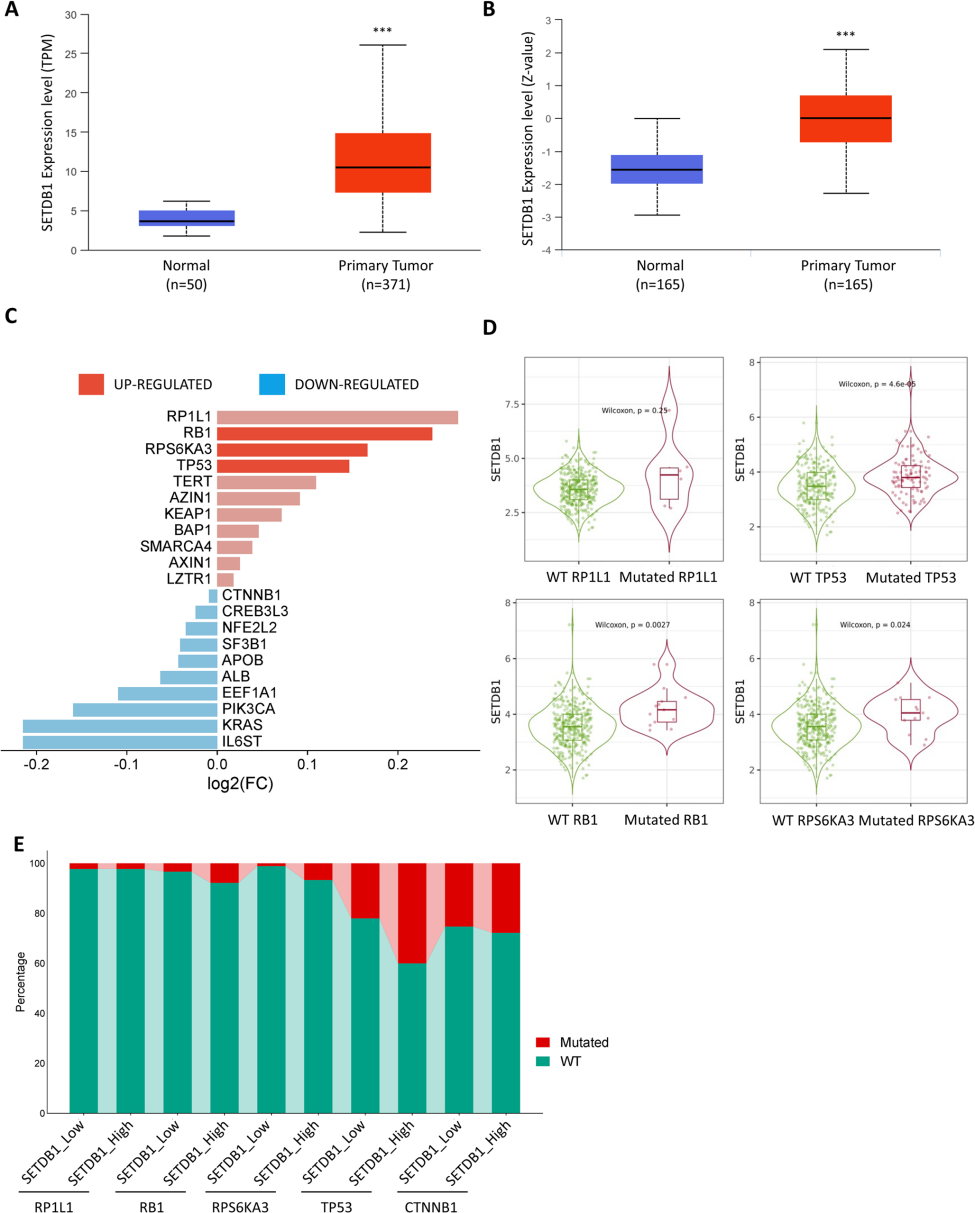
SETDB1 is overexpressed in primary tumors and correlates with specific gene mutations.** (A)** Analysis of relative SETDB1 gene expression (in Transcripts Per Million, TPM) in primary tumor samples (*n* = 371) and adjacent normal tissues (*n* = 50) from the TCGA database. Box plots illustrate the median, interquartile range (IQR), and minimum/maximum values (whiskers). SETDB1 expression is significantly higher in primary tumors. *P* < 0.001. **(B)** SETDB1 protein expression levels (measured in Z-score) in normal (*n* = 165) and primary tumor (*n* = 165) tissues from CPTAC database. The protein levels are also significantly elevated in tumors. *P* < 0.001. **(C)** Bar plot showing correlations between SETDB1 expression and mutation status of driver genes in LIHC. Up-regulation (red) or down-regulation (blue) is indicated by log2 fold change. Significant differences (*P* < 0.05, Spearman’s test) are shown with saturated colors; non-significant (*P* > 0.05) with attenuated colors. **(D)** Violin plots illustrating SETDB1 expression levels in tumors stratified by the mutation status (wild-type vs. mutated) for four key genes: RP1L1, TP53, RB1, and RPS6KA3. Statistical comparisons were performed using a two-sided Wilcoxon rank-sum test. **(E)** A comparison of somatic mutation profiles between groups with high and low SETDB1 expression, defined by the median expression value. The stacked bar plot shows the percentage of mutated (red) and wild-type (green) samples for the top-ranked mutated genes in the TCGA-LIHC cohort

To explore the expression profile of SETDB1 in HCC, we analyzed transcriptomic and proteomic data using the UALCAN online platform. Differential expression analysis revealed a significant upregulation of SETDB1 mRNA in tumor tissues compared to adjacent normal liver tissues (Fig. 1A). In addition, proteomic data from the CPTAC module showed that SETDB1 protein levels were significantly elevated in HCC compared to non-tumoral liver samples (*p* = 1.56E^− 41^) (Fig. 1B).

Given the genomic complexity of HCC, which typically harbors 35 to 80 somatic mutations in coding regions per tumor, we assessed whether SETDB1 expression was associated with key driver mutations. Analysis of TCGA-LIHC data using the Timer2.0 web tool revealed a significant correlation between high SETDB1 expression and frequent mutations in TP53, RB1, and RPS6KA3, with Wilcoxon p-values of 4.6E^− 05^, 0.0027, and 0.0024, respectively (Fig. 1C, D).

To further explore these associations, we stratified patients into SH and SL groups based on median SETDB1 expression. Mutational profiling using the Timer2.0 web tool showed that the SH group exhibited a higher proportion of patients with mutations in these key oncogenes (Fig. 1E). Notably, TP53 mutations were present in approximately 31% of cases and were especially enriched in the SH group. Meanwhile, mutations in RB1 and RPS6KA3 were each found in only 4% of the TCGA-LIHC cohort. These findings suggest that SETDB1 overexpression may be driven, at least in part, by genomic alterations, particularly TP53 mutations, and may play a role in the oncogenic reprogramming of HCC.

**Fig. 2:**
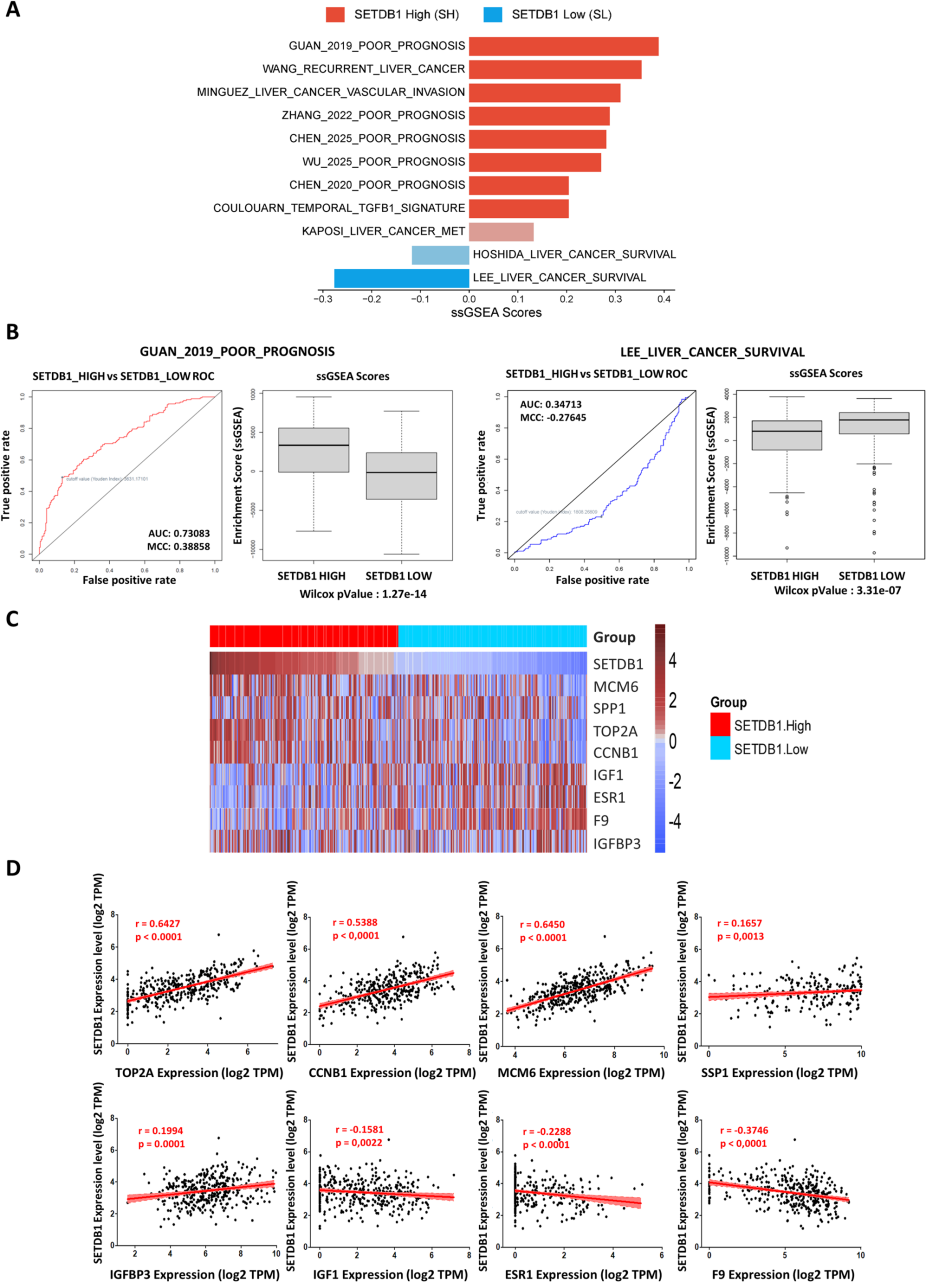
SETDB1 expression is associated with poor prognosis gene signatures.** (A)** Bar plot representing single-sample Gene Set Enrichment Analysis (ssGSEA) enrichment scores of gene signatures related to various adverse clinical outcomes. Positive scores (red bars) indicate an enrichment of the signature in the SETDB1 high expression (SH) group in the TCGA-LIHC cohort, while negative scores (blue bars) indicate enrichment in the SETDB1 low expression (SL) group. Gene sets with a significant enrichment (*P* < 0.05, Wilcoxon test) are shown with saturated colors; non-significant sets (*P* > 0.05) are shown with attenuated colors. **(B)** Receiver Operating Characteristic (ROC) curves and corresponding box plots illustrating the differential enrichment of two specific poor prognosis signatures between the SH and SL groups: “Guan_2019_Poor_Prognosis” and “Lee_Liver_Cancer_Survival”. The Area Under the Curve (AUC) is provided for each ROC curve to assess the predictive power of the signatures. The Wilcoxon P-value for each comparison is also shown. **(C)** Heatmap showing the correlation between SETDB1 expression and the expression of genes from the “Chen_2020_Poor_Prognosis” signature across samples from the TCGA-LIHC cohort. Each row represents a gene from the signature, and each column represents a patient sample. The color intensity corresponds to the expression level (red for high, blue for low). **(D)** Scatter plots illustrating the Spearman’s correlation between SETDB1 expression and the expression of individual genes from the “Chen_2020_Poor_Prognosis” signature. The Spearman’s correlation coefficient (r) and the corresponding P-value are indicated on each plot

To investigate the biological consequences of SETDB1 overexpression, we conducted ssGSEA using curated gene signatures associated with poor prognosis in liver cancer. Our results in TCGA-LIHC showed that the SH group was significantly enriched in gene signatures linked to vascular invasion, early recurrence, and poor survival (Fig. 2A). In contrast, the SL group was associated with favorable survival-related gene sets.

Consistently, receiver operating characteristic (ROC) analysis revealed that gene signatures linked to poor prognosis were upregulated in the SH group, whereas survival-associated signatures were downregulated (Fig. 2B). We also observed that the Coulouarn_temporal_TGFb1_signature, a hallmark of tumor progression and dedifferentiation, was specifically enriched in the SH group, reinforcing the association between SETDB1 expression and aggressive tumor phenotypes. Heatmaps (Fig. 2C) and scatter plots (Fig. 2D) illustrate the correlation between SETDB1 expression and Chen’s eight-gene diagnostic model. SETDB1 expression positively correlates with the TPM values of five genes associated with poor prognosis: *TOP2A*, *CNNB1*, *MCM6*, *SSP1*, and *IGFBP3*, while it negatively correlates with *IGF1*, *ESR1*, and *F9*, which are considered indicative of a good prognosis [25]. These findings were confirmed in the independent validation cohort GSE104580 (Fig. S1A).

Overall, these data demonstrate that high SETDB1 expression is linked to both mutational burden and molecular programs associated with tumor aggressiveness and poor clinical outcomes in HCC. These results highlight the potential of SETDB1 as a prognostic biomarker and suggest its involvement in orchestrating transcriptional programs that drive HCC progression.

### SETDB1 expression correlates with resistance to therapy

The predicted effects of SETDB1 expression on tumor biology do not fully account for the poorer survival outcomes observed in patients with high SETDB1 expression (SH group) within the TCGA-LIHC cohort. Consequently, we investigated whether this unfavorable prognosis could be linked to differences in therapeutic responses, specifically focusing on the impact of SETDB1 expression on sensitivity to immunotherapy, sorafenib, and TACE.

#### SETDB1 expression predicts response to immunotherapy

**Fig. 3:**
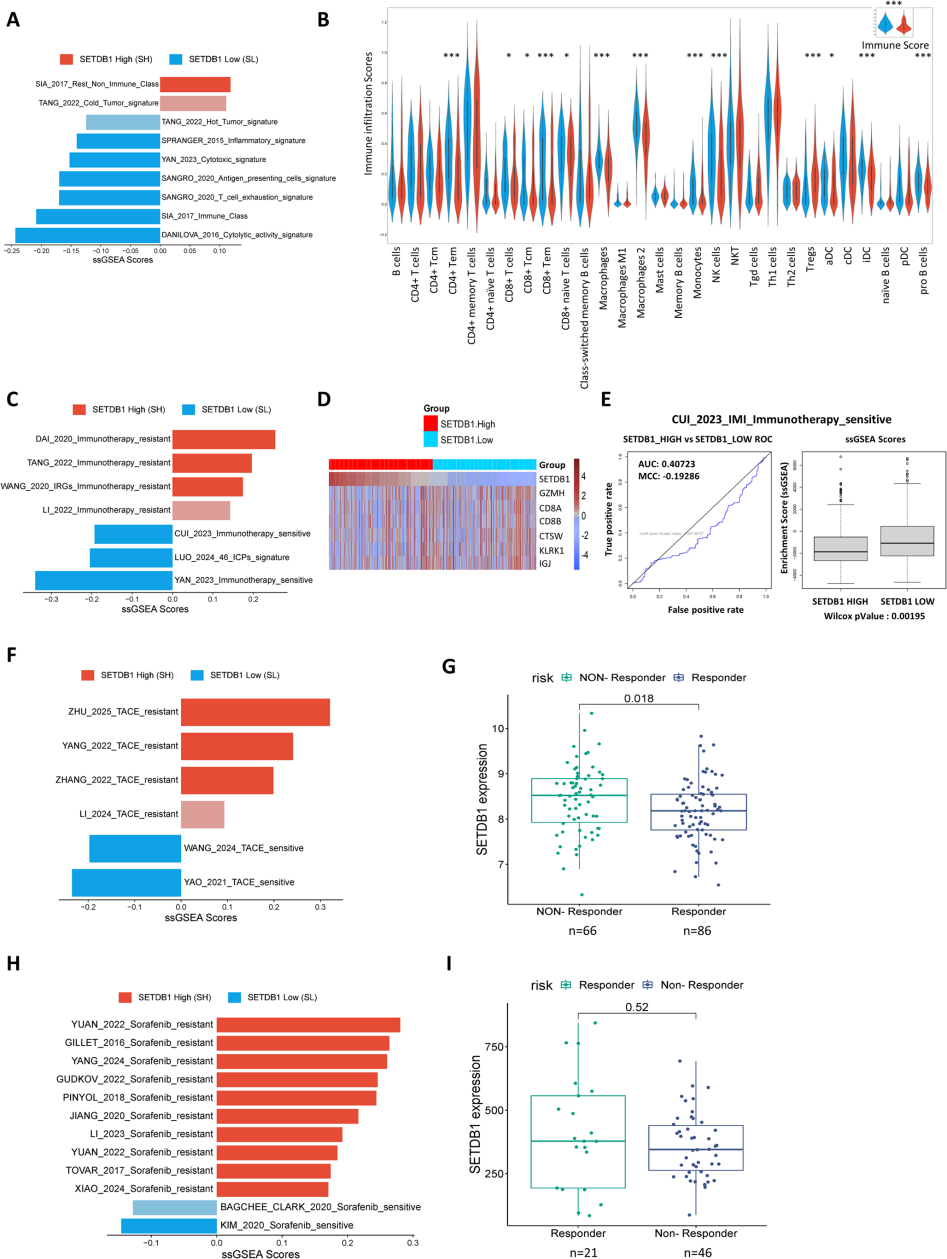
SETDB1 expression predicts response to immunotherapy and systemic therapies in HCC.** (A)** Single-sample Gene Set Enrichment Analysis (ssGSEA) scores of gene signatures related to the immune response, compared between the SETDB1-high (SH) and SETDB1-low (SL) groups in the TCGA-LIHC cohort. Positive scores indicate enrichment in the SH group (red), while negative scores indicate enrichment in the SL group (blue). Saturated colors indicate significant gene sets (*P* < 0.05, Wilcoxon test); attenuated colors are non-significant (*P* > 0.05). **(B)** Violin plots showing the immune cell infiltration scores, as estimated by the XCell algorithm, for 29 immune cell types in SH (red) and SL (blue) groups. *p < 0.05, **p < 0.01, ***p < 0.001, ns = not significant. **(C)** ssGSEA scores of gene signatures related to sensitivity and resistance to immune checkpoint inhibitors (ICIs). Gene sets related to ICI resistance are enriched in the SH group, while those related to sensitivity are enriched in the SL group. **(D)** Heatmap illustrating the correlation between SETDB1 expression and genes from the Immune Index (IMI) signature, a prognostic model for HCC. **(E)** Receiver Operating Characteristic (ROC) curve and box plots evaluating the predictive performance of the IMI signature in distinguishing between SH and SL groups. The Area Under the Curve (AUC) and Wilcoxon P-value are provided. **(F)** ssGSEA scores of gene signatures associated with response to transarterial chemoembolization (TACE). The TACE-resistant signatures are enriched in the SH group. **(G)** Box plot comparing SETDB1 expression levels in TACE responders (*n* = 66) and non-responders (*n* = 86) from the GSE104580 cohort. The P-value from the statistical comparison is shown. **(H)** ssGSEA scores of gene signatures related to sensitivity and resistance to sorafenib. Sorafenib resistance-related signatures are enriched in the SH group. **(I)** Box plot comparing SETDB1 expression levels in sorafenib responders (*n* = 21) and non-responders (*n* = 46) from the GSE109211 cohort. The P-value from the statistical comparison is shown

To characterize the immunological features of the SH and SL groups, we performed ssGSEA using established immune-related gene signatures. SL tumors were significantly enriched in signatures associated with immunogenic and inflamed tumor microenvironments, cytotoxic and antigen-presenting cell signatures, and the inflammatory signature (Fig. 3A). Conversely, the SH group showed enrichment in immune-excluded or non-inflamed tumor phenotypes, highlighting a less immunogenic state. These findings suggest that SETDB1-low tumors resemble “hot tumors” with active immune infiltration, whereas SETDB1-high tumors resemble “cold tumors” with an immunosuppressive microenvironment.

To further explore the immune contexture, we assessed immune infiltration scores using the xCell algorithm. As expected, the immune score was significantly higher in the SL group compared to the SH group (Fig. 3B), confirming a more active immune microenvironment. A detailed analysis of 29 immune cell subtypes revealed that the SL group displayed a greater abundance of antitumoral immune cells, notably CD8 + T cells (*P* < 0.01), CD4 + effector memory T cells, NK cells, M1 macrophages, and dendritic cells, which are known to contribute to effective immune surveillance and tumor rejection. In contrast, the SH group exhibited enrichment of immunosuppressive cells such as Tregs, monocytes, and M2 macrophages, potentially contributing to immune evasion.

Prompted by this strong link between immune features and immunotherapy response, we investigated whether SETDB1 expression influences immunotherapeutic sensitivity. Using ssGSEA, we compared immunotherapy response-related gene signatures between the two groups. The SH group was enriched in gene sets associated with immunotherapy resistance, while the SL group displayed a strong enrichment for signatures predicting sensitivity to immune checkpoint blockade (Fig. 3C). The expression levels of 46 potentially targetable immune checkpoint genes, including the major ones like *PDCD1* (PD-1), *CD274* (PD-L1), and *CTLA4*, were markedly higher in the SL group, further supporting the hypothesis that SETDB1-low tumors are more likely to benefit from immune checkpoint blockade therapy.

To further explore genes linked to immune responsiveness, we examined the CUI_2023_IMI_Immunotherapy_sensitive signature. A heatmap (Fig. 3D) showed a negative correlation between SETDB1 expression and six key IMI genes (*GZMH*, *CD8A*, *CD8B*, *CTSW*, *KLRK1*, and *IGJ*), all associated with robust cytotoxic T/NK cell activity and immunotherapy sensitivity [26]. Enrichment of this signature was higher in the SL group (Fig. 3E), consistent with a greater predicted benefit from immune-based treatments.

Collectively, these results highlight that SETDB1-low tumors exhibit a favorable immunological profile with enhanced infiltration of cytotoxic immune cells and upregulation of immune checkpoint molecules, correlating with a higher likelihood of response to immunotherapies. In contrast, SETDB1-high tumors exhibit immune exclusion and signatures of resistance, which may contribute to their poor prognosis. These results were consistently reproduced in the independent validation cohort GSE104580 (Fig. S1B, C).

#### SETDB1 overexpression is associated with TACE refractoriness

To investigate the association between SETDB1 expression and TACE response, we performed ssGSEA using curated gene signatures in the TCGA-LIHC cohort. Our findings suggest that the SH group exhibits features of TACE refractoriness, as they were significantly enriched in gene sets associated with TACE resistance (Fig. 3F). This association was further confirmed in the GSE104580 cohort (Fig. 3G), where we observed a higher expression of SETDB1 in TACE non-responders compared to responders. These results suggest that SETDB1 expression may be a valuable prognostic biomarker for TACE response in HCC patients, potentially influencing treatment decisions.

#### SETDB1 expression predicts Sorafenib response

We next evaluated the relationship between SETDB1 expression and sorafenib sensitivity, a common therapeutic agent and a tyrosine kinase inhibitor (TKI) used for HCC. Utilizing ssGSEA, we investigated the enrichment of sorafenib resistance-related signatures in both the SH and SL groups (Fig. 3H). The SH group exhibited significant enrichment in gene sets associated with sorafenib resistance, including those linked to multidrug resistance (MDR) [27] and ferroptosis [28], in both the TCGA-LIHC cohort and the GSE104580 validation cohort (Fig. S1D). This suggests that the SH group may exhibit increased resistance to sorafenib treatment due to altered gene expression involved in MDR and ferroptosis pathways. However, we did not observe a significant difference in SETDB1 expression between sorafenib responders and non-responders in the GSE109211 cohort (Fig. 3I). Despite this, a trend suggesting a possible association between SETDB1 expression and sorafenib response was observed, requiring further investigation.

In conclusion, our findings highlight the potential involvement of SETDB1 in mediating therapeutic resistance. These findings suggest that SETDB1 may serve as a valuable biomarker to predict treatment response and guide personalized therapeutic strategies for HCC patients.

### SETDB1 expression defines distinct proliferative, metabolic, and immune transcriptional programs in TCGA-LIHC

ssGSEA and GSEA were performed using the “Hallmarks,” “Kegg_Pathway,” and “Wikipathways” gene set collections to interpret the transcriptomic differences between SH and SL groups.

**Fig. 4:**
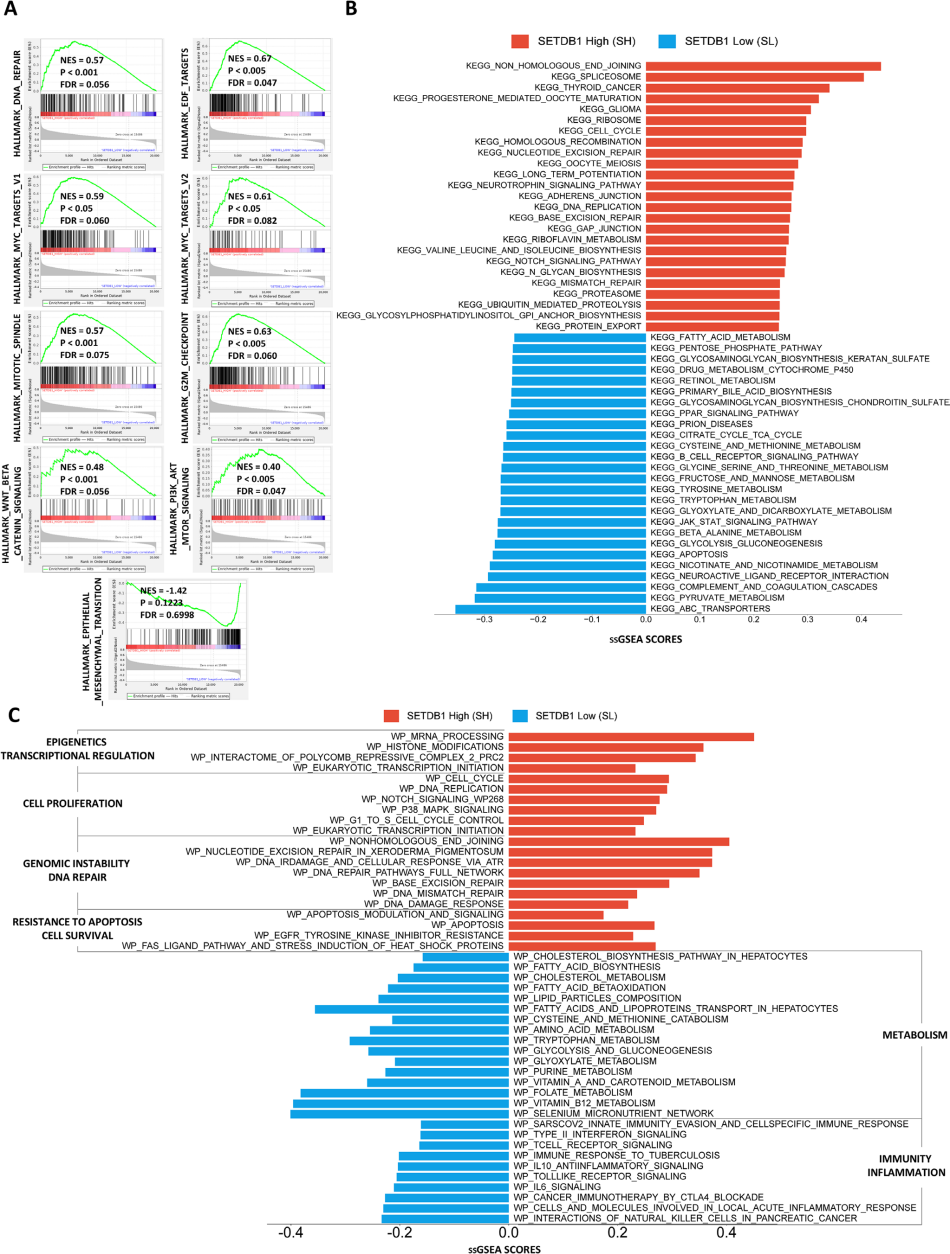
SETDB1 expression is associated with distinct proliferative, metabolic, and immune transcriptional programs in HCC.** (A)** Gene Set Enrichment Analysis (GSEA) plots for six selected gene sets from the Hallmark collection, comparing gene expression profiles between the SETDB1-high (SH) and SETDB1-low (SL) groups. Each plot displays the Normalized Enrichment Score (NES), nominal P-value, and False Discovery Rate (FDR) q-value. Gene sets with an FDR q-value < 0.25 and a NES > 1 (or NES < -1) were considered significantly enriched. **(B)** Bar plot of ssGSEA scores for gene sets from the KEGG collection. Red bars represent pathways enriched in the SH group, while blue bars represent pathways enriched in the SL group. Only pathways with a significant enrichment (*P* < 0.05, Wilcoxon test) are shown. **(C)** Bar plot of ssGSEA scores for gene sets from the WikiPathways collection. The plot is organized by biological function categories, including Epigenetics, Cell Proliferation, Genomic Instability, DNA Repair, Resistance to Apoptosis, Cell Survival, Metabolism, Immunity, and Inflammation. Red bars indicate enrichment in the SH group and blue bars indicate enrichment in the SL group

In the SH group, both ssGSEA and GSEA consistently revealed enrichment for signatures related to cell proliferation, cell cycle progression, and DNA damage repair. Specifically, Hallmarks pathways such as E2F Targets, G2M Checkpoint, Mitotic Spindle, and MYC Targets (V1 and V2) were significantly upregulated (Fig. 4A), suggesting active mitotic processes and oncogenic transcriptional programs. Parallel Kegg_Pathway analysis showed significant enrichment for the cell cycle, DNA replication, and multiple DNA repair pathways (base excision repair, nucleotide excision repair, mismatch repair, and homologous recombination) in SH tumors (Fig. 4B), consistent with enhanced DNA repair capacity. Similarly, Wikipathways analysis revealed an overrepresentation of modules related to DNA damage response and transcriptional regulation (Fig. 4C). These results collectively suggest that high SETDB1 expression is associated with a proliferative, undifferentiated phenotype in HCC, characterized by increased genomic instability and the upregulation of repair and checkpoint mechanisms.

In contrast, the SL group showed significant enrichment for immune-related and metabolic pathways. Immune processes included IL6/JAK/STAT3 signaling, Type I and II Interferon Responses, Toll-like Receptor signaling, and T cell receptor signaling, highlighting a more immunoreactive tumor microenvironment. Metabolic programs, particularly those linked to hepatocytic function (lipid metabolism, amino acid metabolism, and glucose/energy pathways), were also predominant. These findings suggest a metabolically active and well-differentiated hepatic state in the SL group.

In conclusion, the SH group appears to harbor aggressive and proliferative features with elevated DNA repair activity, whereas the SL group displays characteristics of differentiated hepatocytes with active metabolic and immune-competent properties.

ssGSEA and GSEA were performed using the “Hallmarks,” “Kegg_Pathway,” and “Wikipathways” gene set collections to characterize the biological processes associated with SETDB1 expression. These analyses were aimed at interpreting the transcriptomic differences between SH and SL groups.

In the SH group, consistent enrichment for signatures related to cell proliferation, cell cycle progression, and DNA damage repair was revealed by both ssGSEA and GSEA. Specifically, Hallmarks pathways such as E2F Targets, G2M Checkpoint, Mitotic Spindle, and MYC Targets (V1 and V2) were significantly upregulated (Fig. 4A), suggesting active mitotic processes and oncogenic transcriptional programs. In parallel, significant enrichment for the cell cycle, DNA replication, base excision repair, nucleotide excision repair, mismatch repair, and homologous recombination pathways was shown in SH tumors by Kegg_Pathway analysis (Fig. 4B), which is consistent with an enhanced DNA repair capacity. Similarly, an overrepresentation of modules related to DNA damage response and transcriptional regulation was revealed by Wikipathways analysis (Fig. 4C). These results collectively suggest that high SETDB1 expression is associated with increased genomic instability, necessitating the upregulation of repair and checkpoint mechanisms, and supports a proliferative, undifferentiated phenotype in HCC.

In contrast, significant enrichment for immune-related and metabolic pathways was shown in the SL group. The immune processes included IL6/JAK/STAT3 signaling, Type I and II Interferon Responses, Toll-like Receptor signaling, and T cell receptor signaling, highlighting a more immunoreactive tumor microenvironment. Metabolic programs, particularly those linked to hepatocytic function, were also predominant, including lipid metabolism, amino acid metabolism, and glucose/energy pathways. These findings suggest a metabolically active and well-differentiated hepatic state in the SL group.

In conclusion, the SH group appears to harbor aggressive and proliferative features with elevated DNA repair activity, whereas the SL group displays characteristics of differentiated hepatocytes, with active metabolic and immune-competent properties.

### SETDB1 expression drives HCC molecular subclassification

**Fig. 5:**
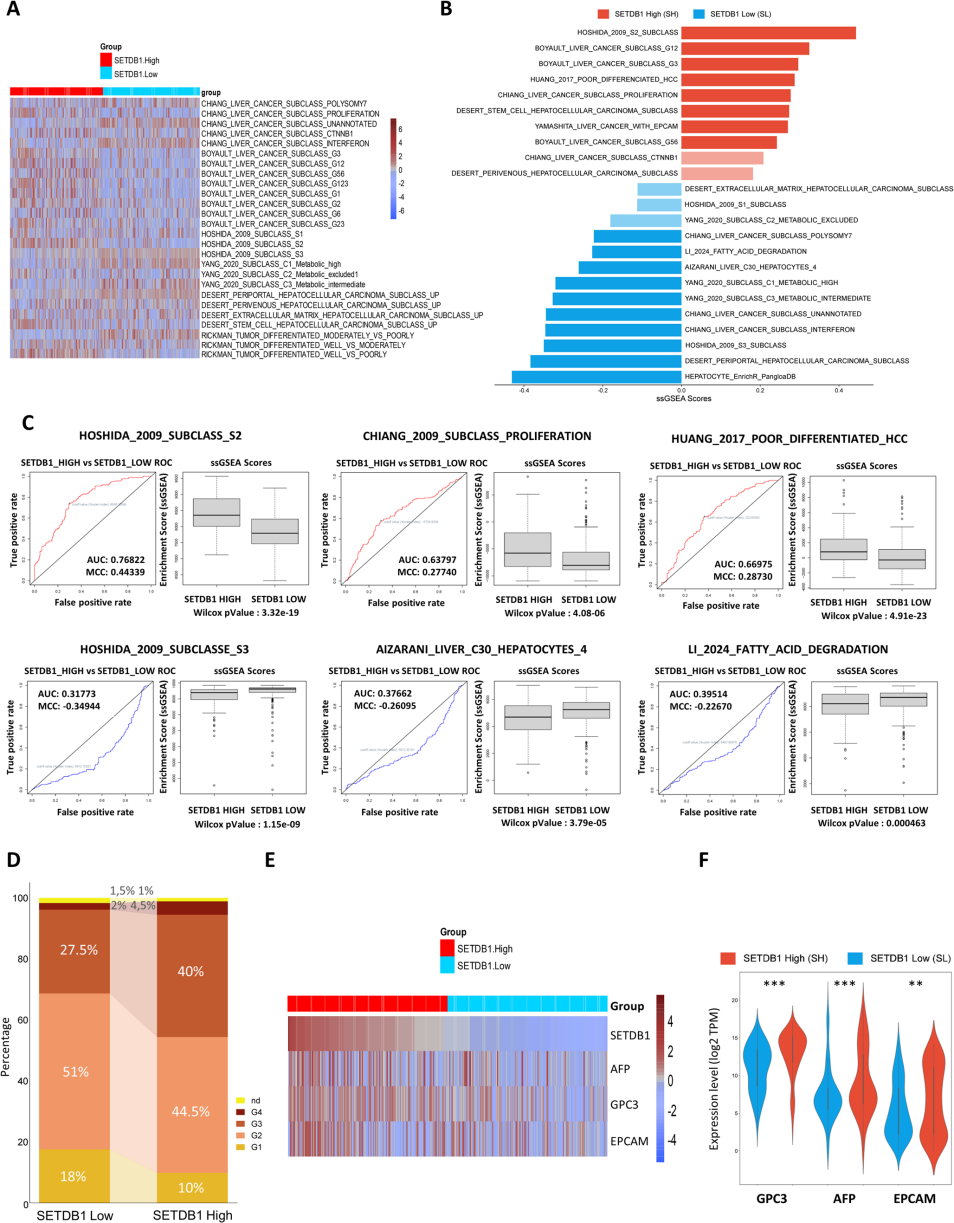
SETDB1 expression is associated with aggressive molecular subtypes and poor differentiation in HCC.** (A)** Heatmap of single-sample Gene Set Enrichment Analysis (ssGSEA) enrichment scores for gene signatures associated with various molecular subclasses and differentiation states of HCC. The heatmap shows that tumors in the SETDB1-high group are primarily enriched for signatures related to aggressive subtypes and poorly differentiated profiles. **(B)** Bar plot of ssGSEA enrichment scores, showing the segregation of SETDB1-high (SH) and SETDB1-low (SL) tumors based on a selection of previously defined HCC molecular subclass signatures in the TCGA-LIHC cohort. Red bars indicate enrichment in the SH group, and blue bars indicate enrichment in the SL group. Saturated colors indicate significant gene sets (*P* < 0.05, Wilcoxon test); attenuated colors are non-significant (*P* > 0.05). **(C)** Receiver Operating Characteristic (ROC) curves and corresponding box plots illustrating the performance of representative signatures in distinguishing between the SH and SL groups. The Area Under the Curve (AUC) and Wilcoxon P-value are provided for each comparison. **(D)** Stacked bar plot showing the distribution of tumor differentiation grades (G1–G4) in the SH and SL groups. This demonstrates a higher proportion of poorly differentiated tumors (G3 and G4) in the SH group. **(E)** Heatmap illustrating the correlation between SETDB1 expression and the expression of three specific markers of poor differentiation and aggressiveness in HCC: AFP, GPC3, and EPCAM. **(F)** Violin plots comparing the expression levels of AFP, GPC3, and EPCAM in the SETDB1-high and SETDB1-low groups. Statistical significance was assessed using the Wilcoxon rank-sum test

To further investigate the role of SETDB1 in shaping HCC intratumoral heterogeneity, we performed a subclass-oriented transcriptomic analysis based on established molecular classification systems. The heatmap (Fig. 5A) illustrates enrichment scores from ssGSEA for various HCC molecular subclasses, as well as signatures of hepatocytic differentiation and metabolic activity, thus providing an integrative view of HCC molecular heterogeneity.

ssGSEA analysis (Fig. 5B, C) showed that the SL group was enriched for subclass signatures linked to well-differentiated, metabolically active HCC, including Hoshida’s S3, Chiang’s interferon subclass, and Désert’s periportal subclass. These subclasses retain hepatocytic identity, display lower mitotic activity, and upregulate metabolic pathways such as the urea cycle, lipid metabolism, and bile acid biosynthesis, consistent with our previous ssGSEA results. The SL group also exhibited enrichment of immune-related pathways, including interferon-γ response, IL6/JAK/STAT3 signaling, and inflammatory signaling, in line with Chiang’s interferon and Hoshida’s S3 classes.

In contrast, the SH group was enriched in dedifferentiated and aggressive molecular subclasses, including Boyault’s G1/G2 and G3, Chiang’s proliferation subclass, Hoshida’s S2, and Désert’s STEM-type. Consistently, the results obtained in the GSE104580 cohort (Fig. S1E) confirmed the molecular subclassification patterns observed in the TCGA-LIHC cohort, although with slightly lower statistical significance.

Overall, the SH group is characterized by poor differentiation, TP53 mutations, chromosomal instability, and activation of oncogenic pathways such as MYC and TGF-beta signaling. In line with our previous observations, the SH group also showed strong activation of cell cycle, DNA repair, MYC targets, and chromatin remodeling programs, alongside a marked downregulation of metabolic pathways typical of Chiang’s proliferation subclass. The SL group was enriched in well-differentiated tumors (grades 1–2) with preserved hepatocytic morphology, whereas the SH group predominated in grade 3 tumors, consistent with a dedifferentiated histopathological profile, higher mitotic activity, and more aggressive clinical behavior (Fig. 5D). In support of these findings, a heatmap (Fig. 5E) shows that the SH group exhibited higher expression of Alpha-fetoprotein (*AFP*), Glypican-3 (*GPC3*), and *EPCAM* (Fig. 5F), all markers associated with dedifferentiation, progenitor-like features, and aggressive behavior. This is further supported by enrichment in Désert’s STEM-type signature.

### SETDB1-high HCC tumors exhibit stemness-associated transcriptional programs, stem cell marker expression, and activation of pluripotency signaling pathways

**Fig. 6:**
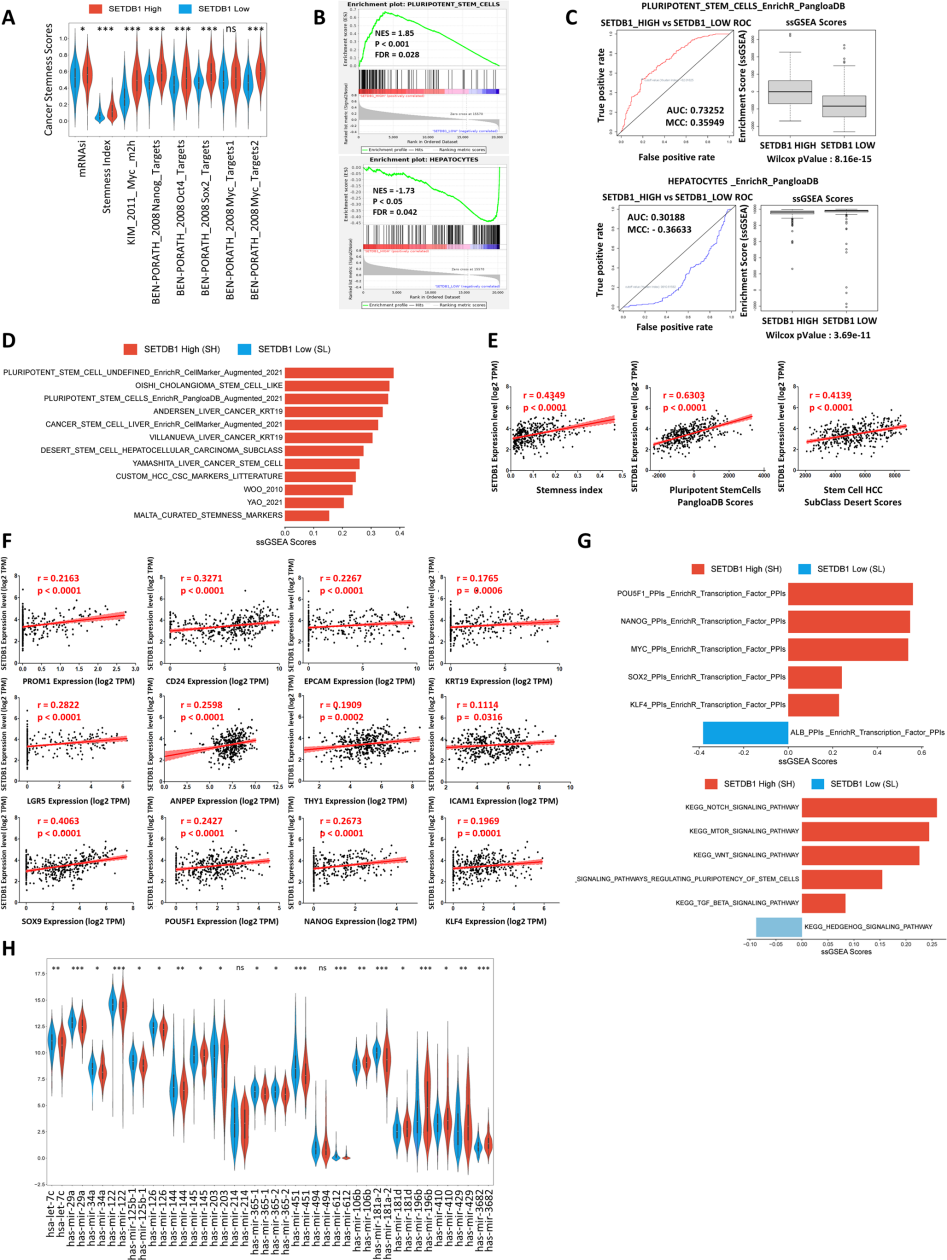
SETDB1 expression is associated with stemness features and a poorly differentiated phenotype in the TCGA-LIHC cohort.** (A)** Violin plots comparing cancer stemness indices (mRNAsi, Stemness Index) between the SETDB1-high (SH) and SETDB1-low (SL) groups. The indices were calculated using the one-class logistic regression model. Statistical significance is indicated: *p < 0.05, **p < 0.01, ***p < 0.001, ns, no significance. **(B)** Gene Set Enrichment Analysis (GSEA) plots demonstrating the enrichment of a pluripotent stem cell signature in the SH group and a hepatocyte signature in the SL group. The Normalized Enrichment Score (NES) and False Discovery Rate (FDR) q-value are shown. **(C)** Receiver Operating Characteristic (ROC) curves and corresponding box plots illustrating the performance of the pluripotent stem cell and hepatocyte signatures in distinguishing between SH and SL groups. The Area Under the Curve (AUC) and Wilcoxon P-value for each comparison are provided. **(D)** Bar plot of single-sample GSEA (ssGSEA) scores showing the enrichment of various stemness-related signatures in the SH (red) and SL (blue) groups. Significant enrichment (*P* < 0.05, Wilcoxon test) is indicated by saturated colors. **(E)** Scatter plots illustrating the Spearman’s correlation between SETDB1 expression and three different stemness scores (mRNAsi, PanglaoDB pluripotency score, and Désert STEM score). The correlation coefficient (r) and P-value are shown for each plot. **(F)** Scatter plots showing the Spearman’s correlation between SETDB1 expression and the expression of ten genes from a custom HCC cancer stem cell (CSC) gene signature. The correlation coefficient (r) and P-value are provided. **(G)** Bar plot of ssGSEA scores for developmental and stemness-related signaling pathways (top) and protein-protein interaction (PPI) modules (bottom). This panel highlights the enrichment of key developmental pathways and pluripotency-related transcription factor modules in the SH group. **(H)** Violin plots comparing the differential expression of selected microRNAs between the SH and SL groups

Following the observation that SETDB1-high tumors are enriched in poorly differentiated and stem-like subclasses (Fig. 5), we investigated whether SETDB1 expression is functionally associated with cancer stemness programs in HCC. Using CancerStemnessOnline, we quantified mRNAsi and StemnessIndex and found both to be elevated in the SH group, indicating a stronger stem-like transcriptional identity (Fig. 6A). To extend these results, GSEA and ssGSEA with the PanglaoDB collection revealed a strong enrichment of pluripotent stem cell signatures in SH tumors, while hepatocyte signatures predominated in the SL group (Fig. 6B, C), highlighting the dichotomy between progenitor-like and hepatocytic differentiation states.

Of note, the SH group showed high expression of *KRT19*, a canonical marker of hepatic progenitors and cholangiocyte-like HCC associated with poor prognosis [29]. In line with this, the SH group exhibited strong enrichment of multiple stemness-related signatures (Fig. 6A and D). *SETDB1* expression positively correlated with mRNAsi, the PanglaoDB pluripotency score, and the Desert STEM score (Spearman *r* = 0.4349, *r* = 0.6303, and *r* = 0.4139, respectively, all *P* < 0.0001) (Fig. 6E). We also found that most genes in a custom HCC CSC signature, including *PROM1* (CD133), *CD24*, *EPCAM*, *KRT19*, *LGR5*, *THY1*, *ICAM1*, *SOX9*, *POU5F1*, *NANOG*, and *KLF4*, were positively correlated with *SETDB1* expression (Fig. 6F), reinforcing its association with progenitor-like states and tumor-initiating potential.

Mechanistically, ssGSEA revealed the activation of multiple developmental and stemness-related signaling pathways in SH tumors, including NOTCH, WNT, mTOR, and TGF-β (Fig. 6G, top). These findings were complemented by the enrichment of protein–protein interaction networks centered on pluripotency transcription factors (*POU5F1* (OCT4), *SOX2*, *NANOG*, *KLF4*, and *MYC*) (Fig. 6G, bottom), suggesting that SETDB1 may act upstream or in concert with these core stemness regulators. Analysis of the GSE104580 cohort (Fig. S1F–H) confirmed the trends in stemness-associated programs observed in TCGA-LIHC. Together, these results support a model in which SETDB1 drives dedifferentiation and stemness in HCC through coordinated transcriptional and signaling networks, fostering intratumoral plasticity and aggressive tumor behavior.

Given the role of non-coding RNAs in regulating differentiation and stemness, we analyzed miRNA profiles in SH and SL tumors. Differential expression analysis identified 5 miRNAs upregulated in the SH group and 13 in the SL group (Fig. 6H). The two groups exhibited contrasting miRNA profiles, with SL tumors enriched in miRNAs linked to differentiation and stemness inhibition (e.g., hsa-miR-612, hsa-miR-365, hsa-miR-126, hsa-miR-34a, hsa-miR-145, hsa-miR-29 family, hsa-miR-144, hsa-miR-203, hsa-miR-451, hsa-miR-122) [26, 30–32], whereas SH tumors showed enrichment of miRNAs promoting stemness (e.g., hsa-miR-196b, miR-429, miR-410, hsa-miR-3682, hsa-miR-181 family, hsa-miR-454) [25, 33, 34]. These findings further reinforce the association between high SETDB1 expression, stemness, and aggressive tumor behavior in HCC.

### SETDB1 expression is predominantly localized in malignant tumor cells and correlates with stemness at the single-cell level

**Fig. 7:**
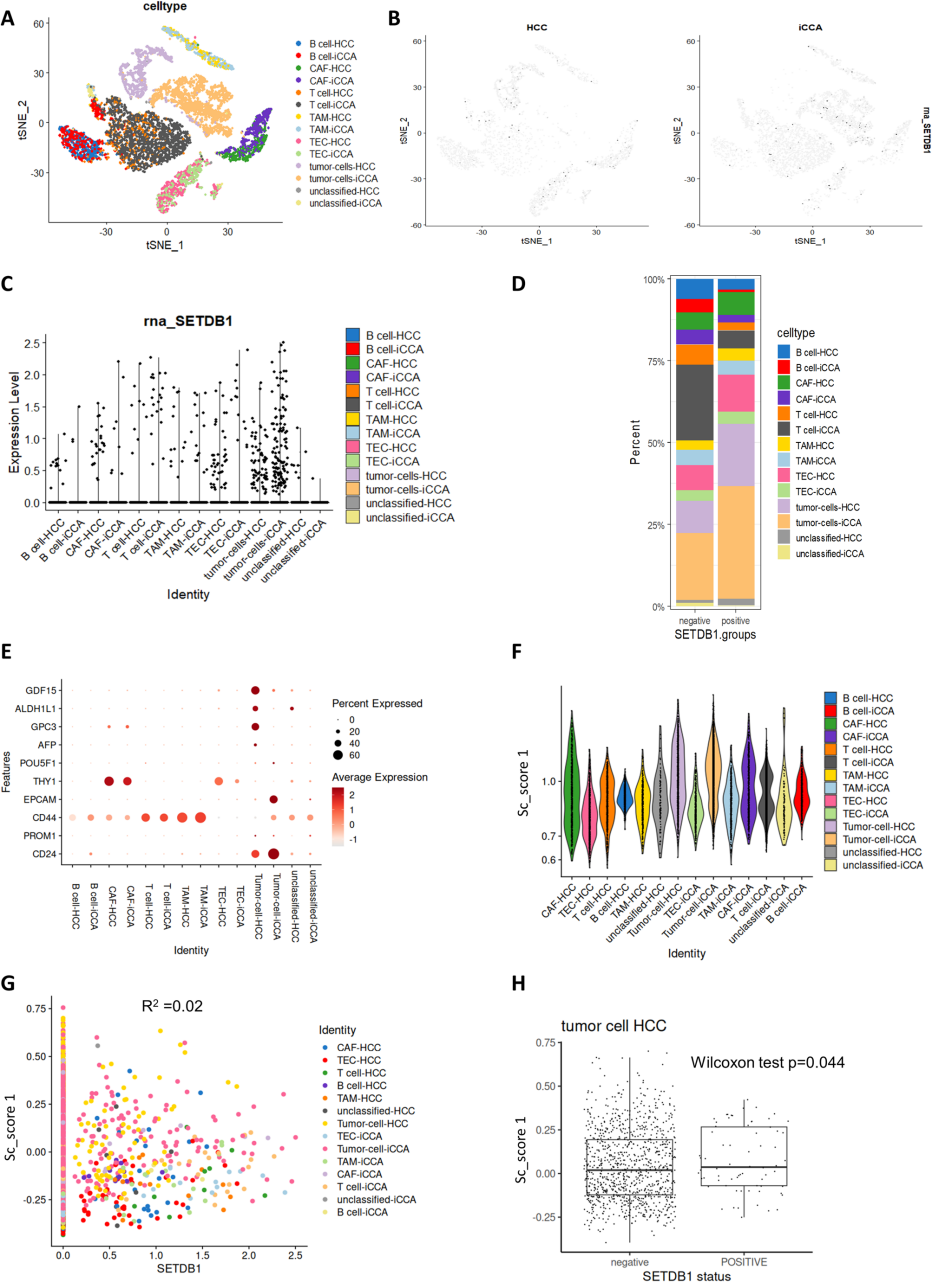
SETDB1 expression is predominantly localized in malignant tumor cells and correlates with stemness at the single-cell level in liver cancer.** (A)** t-SNE dimensional reduction plot of single-cell transcriptomes from liver cancer samples, showing different cell types from HCC (hepatocellular carcinoma) and iCCA (intrahepatic cholangiocarcinoma). Each cluster represents a distinct cell type, as indicated by the color legend. **(B)** t-SNE plots highlighting the expression levels of SETDB1 in HCC and iCCA samples. Darker points correspond to higher SETDB1 expression. This visualization shows that SETDB1 expression is more prominent in tumor cells from both cancer types, with a slightly higher tendency in iCCA tumor cells compared to HCC tumor cells. **(C)** Dot plot showing the expression levels of rna_SETDB1 across different cell types and cancer subtypes. Each dot represents a single cell, and the expression level is plotted on the Y-axis. This figure indicates that SETDB1 is expressed in various cell types but is most frequently expressed in malignant cells (tumor-cells-HCC and tumor-cells-iCCA). **(D)** Stacked bar plot comparing the percentage of different cell types in groups with high (positive) and low (negative) SETDB1 expression. This visualization confirms that the “positive” SETDB1 group is primarily composed of tumor cells. **(E)** Dot plot showing the expression of stemness marker genes (GDF15, ALDH1L1, GPC3, AFP, EPCAM, CD44, and CD24) across diverse cell populations, including tumor cells, cancer-associated fibroblasts (CAFs), tumor endothelial cells (TECs), tumor-associated macrophages (TAMs), B cells, T cells, and unclassified populations. Dot size represents the percentage of cells expressing each gene, while color intensity reflects the average expression level. **(F)** Violin plots depicting the distribution of stem cell score (sc_score1) across different cell identities, showing enrichment in tumor cell populations. **(G)** Scatter plot illustrating the positive correlation between SETDB1 expression and stem cell score (sc_score1) in HCC tumor cells. Each point represents an individual cell, with color indicating cell identity. **(H)** Box plot comparing stem cell scores (STEM/CELL_score) between SETDB1-positive and SETDB1-negative HCC tumor cells. SETDB1-positive cells exhibit significantly higher stemness scores (Wilcoxon test, *p* = 0.044)

Single-cell transcriptomes from 9 HCC and 10 iCCA samples in dataset GSE125449 [24] were investigated using the Seurat R package (version 4.3.0) [35] in the R software environment (version 4.2.1). After prefiltration (genes expressed in over 100 cells and genes with minimal expression in at least 3 cells), a total of 9946 single-cell transcriptomes were normalized, scaled, and subjected to dimension reduction by tSNE method (Fig. 7A).

Expression of SETDB1 was investigated by tSNE (Fig. 7B) and volcano plot (Fig. 7C) visualization after stratification by LIHC subgroups and the cellular types present in tumors and their microenvironment. We observed that SETDB1 is expressed in different cell types in both subtypes of liver cancer (Fig. 7C, D). A more frequent expression of SETDB1 was found in malignant cells from both cancer types, with a slightly higher tendency in iCCA-derived cells compared to HCC ones. These results indicate that SETDB1 expression is primarily found in malignant tumor cells and is less prevalent in microenvironmental cellular subpopulations.

Single-cell transcriptomic analysis revealed distinct patterns of stemness marker gene expression across tumor and stromal cell populations in HCC and iCCA. Stemness-associated genes such as GDF15, ALDH1L1, GPC3, AFP, EPCAM, CD44, and CD24 were predominantly expressed in tumor cells, with varying levels across other cell types including CAFs, TECs, and TAMs (Fig. 7E). Consistently, the stem cell score (sc_score1), computed from the expression of these markers, was significantly higher in tumor cells compared to stromal and immune populations, indicating enrichment of stem-like transcriptional programs (Fig. 7F). Given this enrichment, we next investigated whether SETDB1 expression was associated with stemness at the single-cell level. Across all cell populations, SETDB1 expression showed only a weak association with stem cell score (R² = 0.02), reflecting *substantial* cellular heterogeneity and the absence of a global linear relationship (Fig. 7G). Importantly, when focusing specifically on HCC tumor cells, SETDB1-positive cells exhibited significantly higher stem cell scores compared to SETDB1-negative counterparts (Fig. 7H, Wilcoxon *p* = 0.044), supporting the association between SETDB1 expression and enhanced stem-like properties in HCC liver cancer cells.

### Hypoxia increases SETDB1 expression and stemness, while paclitaxel treatment leads to their suppression

**Fig. 8:**
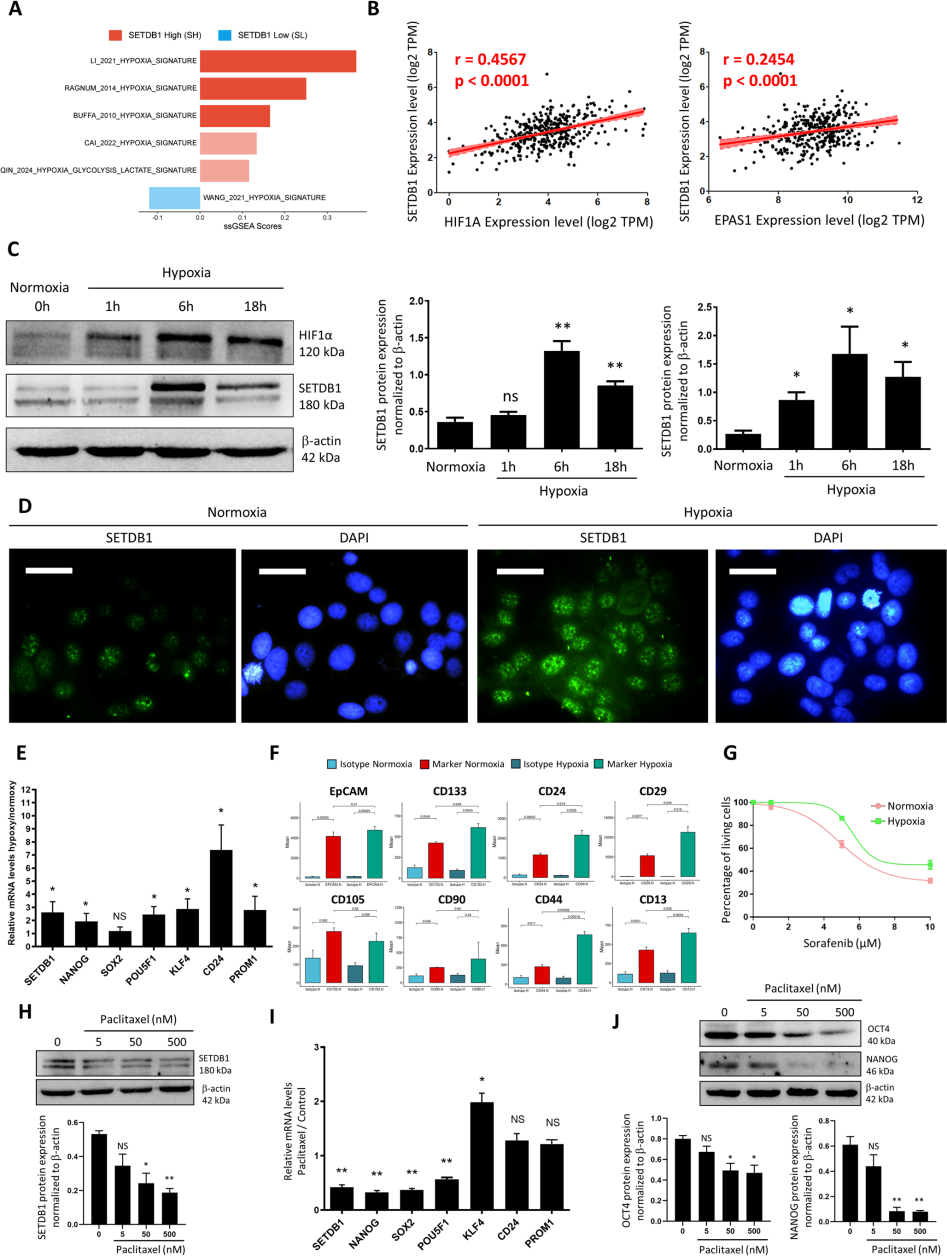
SETDB1 expression mirrors stemness dynamics in HuH7WT cells under hypoxia and paclitaxel treatment.** (A)** ssGSEA scores of hypoxia-related gene signatures in SH and SL groups. The bar graph shows the enrichment of hypoxia-related gene sets. Red bars indicate enrichment in the SH group, and blue bars indicate enrichment in the SL group. Saturated colors indicate significant gene sets (*P* < 0.05, Wilcoxon test); attenuated colors are non-significant (*P* > 0.05). **(B)** Scatter plots showing Spearman’s correlation between SETDB1 expression and hypoxia-inducible factor genes (left, HIF1A and right, EPAS1, HIF-2α) in the TCGA-LIHC cohort. **(C)** Western blot analysis of SETDB1 and HIF-1α protein expression under hypoxic conditions. The blot and corresponding bar graph show the expression of SETDB1 and HIF-1α in Huh7 cells after 6 h of hypoxia compared to normoxia. β-actin served as the internal loading control. Data are represented as means ± SE, *n* = 3. Statistical significance is indicated as follows: *P < 0.05, **P < 0.01, ***P < 0.001, ****P < 0.0001. **(D)** Immunofluorescence analysis of SETDB1 localization under hypoxic conditions. The images show nuclear (DAPI, blue) and cytoplasmic/nuclear (SETDB1, green) staining of SETDB1 in Huh7 cells under normoxic and 6-hour hypoxic conditions. Data are represented as means ± SE, *n* = 3. Scale bar = 200 μm. **(E)** qPCR analysis of stemness markers in Huh7 cells after 24-hour hypoxia, showing relative mRNA levels of *NANOG*, *SOX2*, *POU5F1*, *KLF4*, *CD24*, and *PROM1* normalized to 18 S and expressed versus normoxia. **(F)** Flow cytometry of stemness-associated surface markers (CD133, CD24, CD44) in Huh7 cells after 3 days of hypoxia versus normoxia. **(G)** Sorafenib sensitivity assay under hypoxic conditions. The graph shows the dose-response curve of sorafenib in Huh7 cells cultured under normoxic and 3-day hypoxic conditions, highlighting the change in IC50. **(H)** Western blot of SETDB1 protein expression SETDB1 in Huh7 cells treated with increasing concentrations of paclitaxel (5-500 nM, 48 h). The blot and bar graph depict SETDB1 protein levels, with β-actin as loading control. **(I)** qPCR analysis of stemness markers in Huh7 cells treated with paclitaxel (500 nM, 24 h), showing relative mRNA levels normalized to 18 S and expressed versus control. **(J)** Western blot of OCT4, and NANOG protein expression SETDB1 in Huh7 cells treated with increasing concentrations of paclitaxel (5-500 nM, 48 h). The blot and bar graph depict SETDB1 protein levels, with β-actin as loading control

Hypoxia is a well-known inducer of stemness in LIHC, playing a crucial role in driving metabolic shifts, cancer progression, and therapeutic resistance. Given this, we assessed the relationship between SETDB1 expression and hypoxia. We performed ssGSEA using curated hypoxia-related signatures (Buffa and Ragnum, validated across multiple cancer types [36]) in the TCGA-LIHC cohort. Our analysis showed that SH tumors displayed pronounced hypoxic features (Fig. 8A). Consistently, *SETDB1* expression correlated positively with *HIF1A* and *EPAS1* (HIF-2α) expression (Fig. 8B), reinforcing its association with hypoxia in HCC.

To validate these findings in vitro, we exposed Huh7 and HepG2 cells to hypoxic conditions (1% O2) for 3–48 h. Western blot revealed a marked upregulation of SETDB1 and HIF-1α protein expression in Huh7 cells after 6 h of hypoxia (Fig. 8C). Moreover, immunofluorescence showed weak nuclear SETDB1 staining in normoxia, which became markedly stronger after 6 h of hypoxia (Fig. 8D), further reinforcing the role of hypoxia in modulating SETDB1 expression. Building on these results, qPCR demonstrated elevated *SETDB1* mRNA along with pluripotency markers (*NANOG*, *POU5F1*, *SOX2*, *KLF4*, *CD24*, and *PROM1*) in Huh7 cells after 24 h of hypoxia (Fig. 8E). Flow cytometry analysis confirmed increased stem-like markers (CD133, CD24, and CD44) in Huh7 cells after 3 days of hypoxia (Fig. 8F). To explore the functional implications of these stemness traits, we assessed the sensitivity of Huh7 cells to Sorafenib. Sorafenib sensitivity assays showed an increased IC50 from 4.88 µM (normoxia) to 5.47 µM (3 days hypoxia) (Fig. 8G), suggesting enhanced therapeutic resistance.

Furthermore, to evaluate the relationship of SETDB1 levels and stemness gene expression, we used paclitaxel (taxol), a described SETDB1 inhibitor [37]. Western blot revealed a marked downregulation of SETDB1 upon paclitaxel treatment at both 50 nM and 500 nM (Fig. 8H). This decrease in SETDB1 inhibition was correlated with reduced mRNA levels of three key stemness transcription factors (*NANOG*,* SOX2*,* and POU5F1*) (Fig. 8I). At the protein level, NANOG, and to a lesser extent OCT4, was also decreased under paclitaxel at 50 nM and 500 nM. Experiments performed with HepG2 cells were concordant with those in Huh7cells, showing that hypoxia increased both SETDB1 and stemness markers, whereas paclitaxel treatment was associated with their reduction (Fig. S2A-D).

Collectively, these results support a strong association between SETDB1 and stemness traits under hypoxia, including elevated expression of stem cell markers and reduced drug sensitivity. Conversely, lower SETDB1 levels were observed alongside a less pronounced stem-like phenotype, indicating a close correlation between SETDB1 and stemness features.

### SETDB1 expression and its chromatin binding partners are associated with poor disease-free survival in liver cancer

**Fig. 9:**
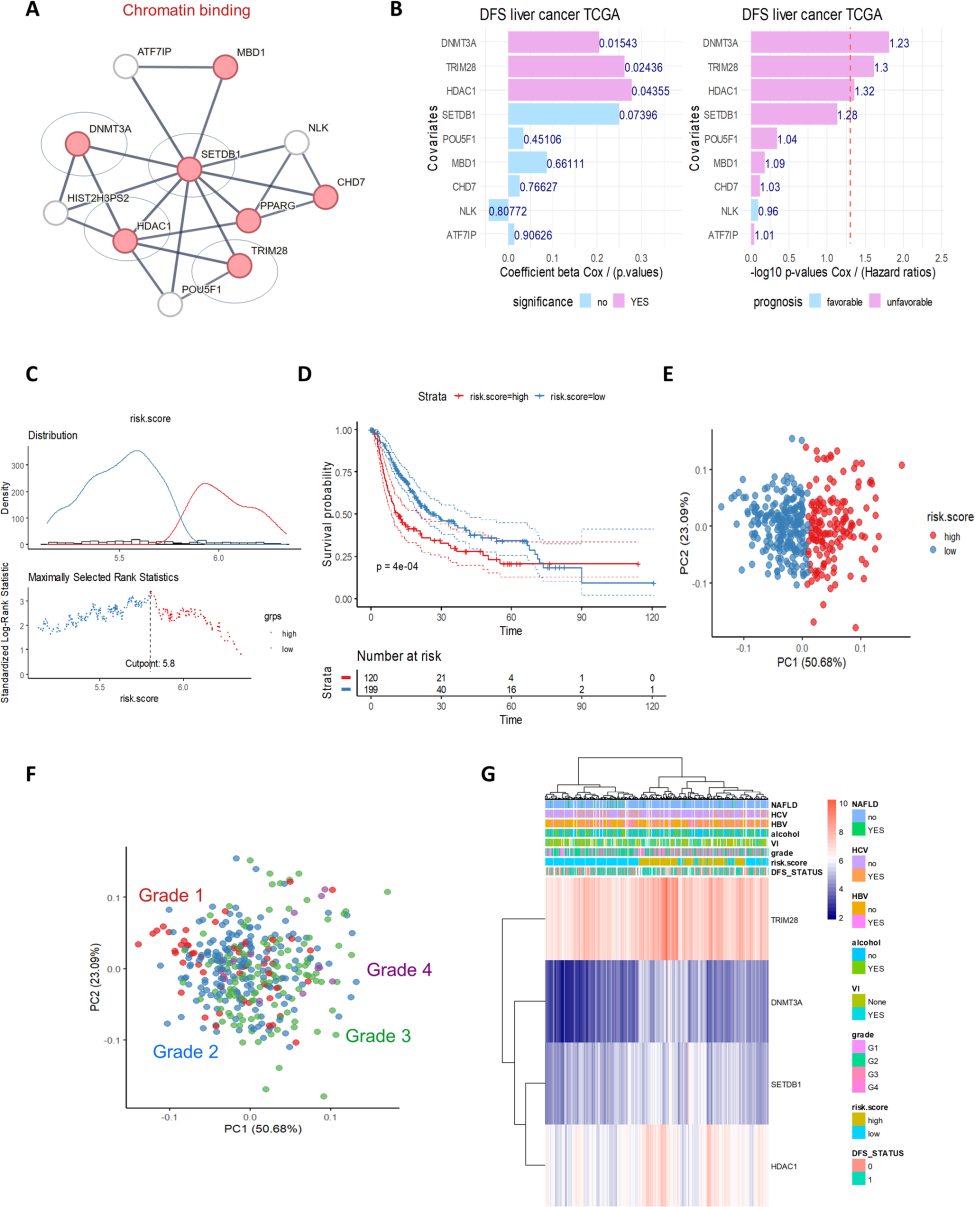
A SETDB1-Related Expression Risk Score Stratifies HCC Patients in the TCGA Liver Cancer Cohort.** (A)** Protein-protein interaction (PPI) network of SETDB1 and its partners. The network, analyzed using the STRING server, highlights SETDB1’s chromatin-binding partners (red nodes) used to calculate the expression risk score. **(B)** Univariate Cox analyses for disease-free survival (DFS). The forest plot shows the coefficient beta and -log10 p-values for each marker, highlighting partners with a significant correlation to prognosis. The dashed red line indicates a hazard ratio of 1.0. **(C)** Risk score distribution and optimal cutpoint determination. The upper panel shows the distribution of the risk score, while the lower panel shows the determination of the optimal cutoff threshold using standardized log-rank statistics, which stratifies patients into low- and high-risk groups. **(D)** Kaplan-Meier survival curves for DFS. The plot shows DFS stratified by the low- and high-risk score categories. The table below the graph indicates the number of patients at risk over time. **(E)** Principal component analysis (PCA) plot stratified by risk score. The plot is based on the expression of SETDB1 and its partners (*TRIM28*, *HDAC1*, and *DNMT3A*). Patients are colored according to their risk score (low risk in blue, high risk in red), showing a clear separation of the groups. **(F)** PCA plot stratified by pathological grade. The same patient cohort is stratified by pathological grade (Grade 1 to Grade 4), indicating that the expression of these markers correlates with tumor differentiation. **(G)** Unsupervised hierarchical clustering and heatmap analysis. The heatmap displays the expression levels of the SETDB1-related risk score markers. The top annotation bar includes key clinical variables, such as viral status (HCV, HBV), vascular invasion (VI), and pathological grade, showing that the clustering of high-risk samples corresponds to high-grade tumors

To investigate the prognostic role of SETDB1 and its partners in liver cancer, their PPI network was analyzed using the STRING server. The resulting network (Fig. 9A) contained eleven molecules, including SETDB1, and its functional enrichment revealed a significant enrichment for chromatin binding (FDR *p* = 1.8 × 10^− 5^).

Prognostic analyses identified three markers with a significant positive association with poor disease-free survival (DFS) (univariate *p* < 0.05): DNMT3A (hazard ratio = 1.23, *p* = 0.015), TRIM28 (hazard ratio = 1.30, *p* = 0.024) and HDAC1 (hazard ratio = 1.32, *p* = 0.043). The SETDB1 hazard ratio was 1.28 (*p* = 0.074) (Fig. 9B, Table S4). These four prognostic markers are all involved in chromatin binding.

A risk score was then computed for the TCGA liver cancer cohort using the expression of these four genes. Patients were stratified into high- and low-risk groups for DFS using an optimal threshold (cutpoint: 5.8). A significant stratification of patients based on this risk score was confirmed by Kaplan-Meier survival analysis and a log-rank test (univariate *p* = 4 × 10^− 4^) (Fig. 9C).

A clear separation of the high- and low-risk groups was also demonstrated by PCA based on the expression of the four binders (Fig. 9D). Interestingly, samples were also stratified by pathological grade in this same PCA (Fig. 9E). Furthermore, high-risk samples with high-grade values were successfully aggregated by unsupervised clustering of these four chromatin binders’ expression profiles (Fig. 9F). No association was observed between the clustering and other clinical risk factors (e.g., vascular invasion, hepatitis B or C, NAFLD, or alcohol consumption) (Fig. 9G).

**Fig. 10:**
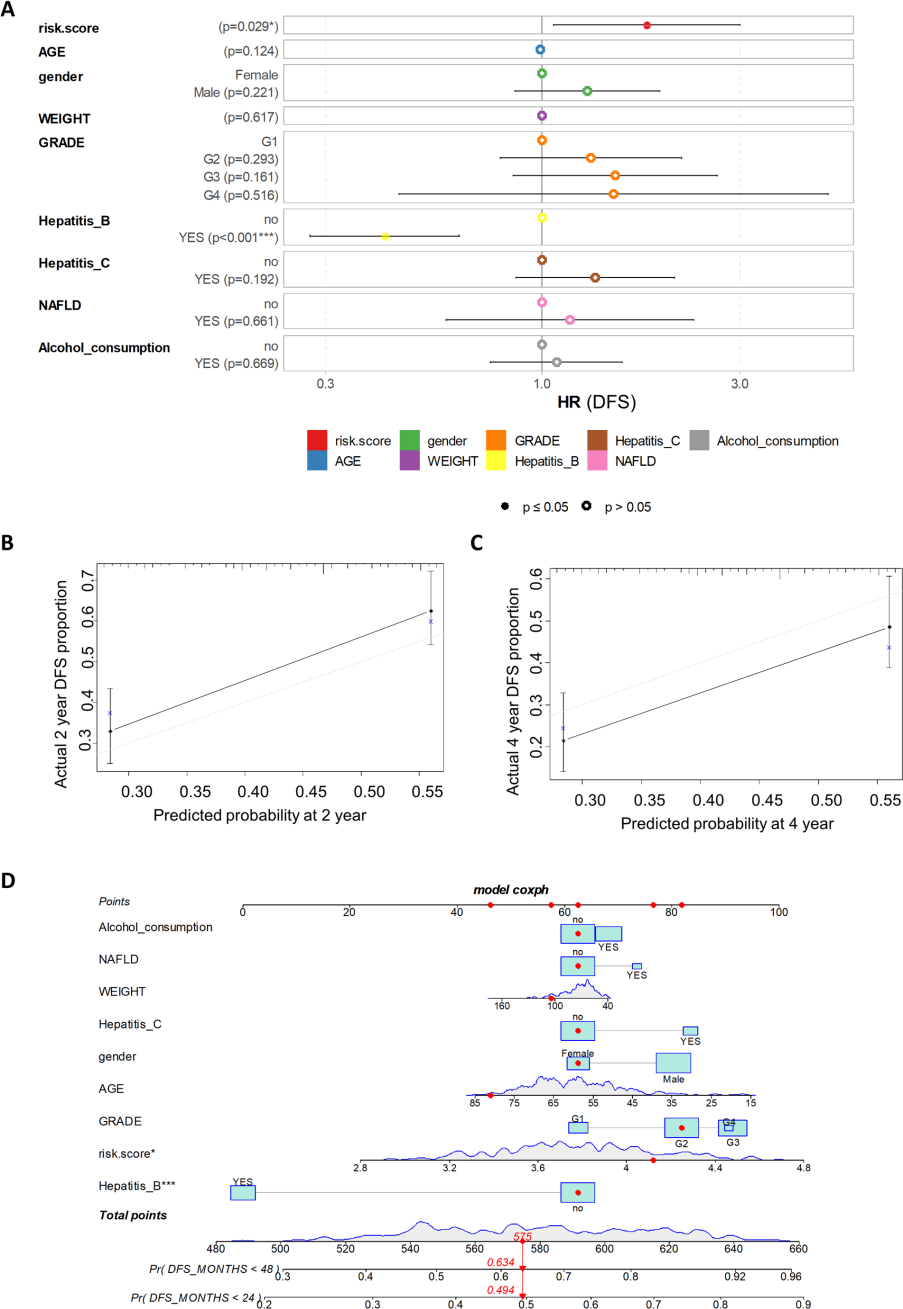
The SETDB1-Related Risk Score is an Independent Unfavorable Prognostic Factor for Disease-Free Survival in Liver Cancer.** (A)** Multivariable Cox model for disease-free survival (DFS) in the TCGA cohort. The forest plot shows the hazard ratio (HR) and p-values for the SETDB1-related risk score and various clinical and demographic factors, including age, gender, weight, pathological grade, and liver cancer etiology (hepatitis B, hepatitis C, NAFLD, and alcohol consumption). **(B)** Calibration plot of the multivariable DFS model at two years. This plot, performed by bootstrapping, shows the predicted probability of disease-free survival at two years compared to the observed outcome, indicating the model’s predictive accuracy. **(C)** Calibration plot of the multivariable DFS model at four years. Similar to panel B, this plot shows the model’s predictive performance for the probability of disease-free survival at four years. **(D)** Nomogram for predicting DFS. The nomogram illustrates the probability of disease-free survival at two and four years of follow-up. The red markers highlight the values for a representative individual case from the TCGA cohort

To assess the independence of the SETDB1-related risk score, a multivariable Cox model for DFS was constructed. The risk score, demographic parameters (age, gender, weight), grade, and risk factors were included in this model. The model was significant (log-rank *p* = 0.001, Fig. 10A), and the SETDB1-related risk score was confirmed as an independent unfavorable prognostic factor for DFS (multivariable hazard ratio = 1.78, *p* = 0.029).

The model’s reliability was then validated through bootstrapping (500 iterations) to predict the probability of DFS at two and four years (Fig. 10B, C). Both calibrations showed that the model has good predictive power. The nomogram highlighted the significant weight of the SETDB1 risk score in predicting patient outcomes (Fig. 10D). These findings suggest that SETDB1 and its chromatin binding partners (DNMT3A, HDAC1, and TRIM28) are deregulated during liver cancer progression. Their combined expression serves as an independent prognostic factor for poor disease-free survival, indicating their potential as a valuable biomarker for patient stratification.

## Discussion

HCC remains a major global health challenge due to its increasing incidence, high mortality, and a persistent lack of effective long-term therapeutic strategies. Despite advances in locoregional and systemic treatments, including TACE, tyrosine kinase inhibitors (TKIs), and immune checkpoint inhibitors (ICIs), durable responses remain rare, in part because of the profound intratumoral heterogeneity and the persistence of CSCs [38]. These CSCs possess remarkable adaptive capabilities, enabling them to withstand therapeutic pressures through mechanisms such as enhanced DNA repair, ABC transporter activation, metabolic rewiring, and suppression of apoptosis. They further contribute to an immunosuppressive tumor microenvironment via intrinsic mechanisms (PD-L1 upregulation, TGF-β secretion) and extrinsic modulation (recruitment of regulatory T cells, M2 macrophages) [39], ultimately sustaining tumor survival, recurrence, and metastasis. Therefore, identifying upstream regulators of cancer stemness and effectively targeting CSCs are critical priorities for improving patient outcomes. Among the epigenetic regulators implicated in HCC, the histone methyltransferase SETDB1 emerges as one of the most upregulated and functionally relevant [40–43]. Beyond its well-established role as a transcriptional repressor implicated in tumorigenesis and immune evasion [40], the specific contribution of SETDB1 to liver CSC regulation had remained largely unexplored. Through integrative multiomic analyses with hallmark, KEGG, and curated stemness gene signatures, we highlight SETDB1 as a defining feature of a stemness-enriched, poorly differentiated, clinically aggressive HCC phenotype. Single-cell RNA-seq analyses revealed that SETDB1 expression is largely confined to malignant epithelial cells, with marked enrichment in progenitor-like subsets (EPCAM⁺, PROM1⁺, ALDH1A1⁺, KRT19⁺, CD24⁺) previously linked to hepatic progenitor cells and CSC-like features [24]. Consistent with these findings, bulk TCGA-LIHC tumors stratified by SETDB1 expression revealed a clear dichotomy. SETDB1-low (SL) tumors retained hepatocytic differentiation, exhibited low proliferative indices, and were enriched in favorable-prognosis molecular subclasses, whereas SETDB1-high (SH) tumors aligned with aggressive subclasses characterized by strong MYC activation, cell-cycle progression, TGF-β signaling, and a marked loss of hepatocytic identity.

In addition, SETDB1-high tumors displayed a significantly higher burden of TP53 mutations, most notably the R249S hotspot. Mutant TP53 has been shown to enhance SETDB1 transcription and protein stability [44], fostering cell cycle progression and apoptosis evasion [45], a feedback loop that may fuel aggressive, therapy-resistant clones. The tumor microenvironment also contributes to SETDB1 accumulation, particularly through hypoxia, a well-established driver of CSC maintenance. Acting via hypoxia-inducible factors (HIFs), metabolic rewiring, and epigenetic regulation, hypoxia sustains CSC properties and promotes tumor progression, therapy resistance, and recurrence. Our transcriptomic analyses and in vitro data demonstrate that hypoxic stress markedly increases SETDB1 expression. Mechanistic experiments have shown that, under low-oxygen conditions, SETDB1 evades CRL2^VHL^-mediated proteasomal degradation, leading to its stabilization, as the protein escapes the oxygen-dependent activity of this E3 ubiquitin ligase complex [46]. Adding to these layers of regulation, SETDB1 is also integrated into an oncogenic feed-forward loop with MYC, wherein MYC promotes SETDB1 expression and SETDB1 reinforces MYC-driven transcriptional programs [47]. This feedback architecture may help stabilize a dedifferentiated, stemness-dominated cellular state that confers high proliferative potential and therapeutic resistance. Collectively, the convergence of TP53 mutations, hypoxia-driven stabilization, and MYC-mediated transcriptional reinforcement provides a compelling explanation for why SETDB1 is selectively overexpressed in the most aggressive HCC subtypes.

Beyond the upstream factors driving SETDB1 overexpression, its functional impact on CSC regulation is mediated through several interconnected epigenetic and transcriptional mechanisms. The strong correlation between SETDB1 expression and multiple independent stemness signatures suggests that SETDB1 is embedded within a broader pluripotency-associated regulatory network. This is consistent with evidence from embryonic stem cells demonstrating that SETDB1 is essential for maintaining pluripotency and early embryonic viability. Indeed, SETDB1-deficient embryos exhibit peri-implantation lethality, with developmental arrest around E4.5 [11], and SETDB1 depletion in vitro prevents the formation of stable ESC colonies [11]. Mechanistically, SETDB1 sustains pluripotency through two complementary modes of action. First, it physically interacts with OCT4, and loss of SETDB1 reduces OCT4 expression [48, 49], alters colony morphology, and triggers differentiation programs. Second, SETDB1 deposits the repressive H3K9me3 mark on differentiation-associated genes, thereby silencing their expression and indirectly stabilizing the pluripotent state [50]. For example, Wu et al. demonstrated that SETDB1 represses the totipotency factor Dux, preventing activation of 2 C-like programs and ensuring the transition from totipotency to pluripotency [51]. Together, these mechanisms highlight a conserved role for SETDB1 in maintaining stemness, a function that CSCs appear to exploit in oncogenic contexts. SETDB1 also supports CSC traits through its interaction with PML nuclear bodies (PML-NBs), non-membranous nuclear structures that coordinate chromatin modifiers, transcriptional regulators, and DNA repair factors essential for chromatin organization and CSC maintenance. By stabilizing PML-NBs, SETDB1 preserves the nuclear scaffold required for assembling repressive chromatin domains, whereas its inhibition disrupts these compartments [52]. Given that PML degradation reduces OCT4 levels and depletes CSCs [53, 54], SETDB1 may promote stemness not only through H3K9me3-mediated repression but also by stabilizing PML-NB–dependent nuclear architectures that preserve stem cell identity.

Among the targets silenced through these combined mechanisms, SETDB1 represses endogenous retroelements (ERVs) under hypoxic conditions, preventing the accumulation of transposon-derived dsRNAs that would otherwise activate innate immune pathways and induce lethal DNA damage [55]. By maintaining ERV silencing, SETDB1 acts as a genome-protective factor that enables CSCs to withstand oxygen deprivation, metabolic stress, and immune pressure. This transposon-silencing function, previously shown to be essential for preserving stem cell identity and genomic stability [56], likely contributes to CSC maintenance in hostile microenvironmental niches.

SETDB1 further contributes to HCC heterogeneity by modulating miRNA programs that govern stemness and differentiation [57]. SH tumors show upregulation of oncogenic miRNAs (e.g., miR-196b, miR-181 family, miR-454-3p) that promote CSC features and resistance [25, 33, 34], while tumor-suppressive miRNAs (e.g., miR-29a/b, miR-122) are downregulated [26, 30, 31]. This dual regulation suggests that SETDB1 reinforces tumor plasticity not only through chromatin remodeling but also by orchestrating a pro-oncogenic miRNA environment. This is further supported by the downregulation of miRNAs that directly target the SETDB1 3′-UTR in SH tumors [43, 58], contributing to SETDB1 overexpression, CSC enrichment, and therapeutic resistance.

SETDB1 may also contribute to the regulation of CSCs through EMT. Several studies have shown that SETDB1 promotes EMT [59, 60], tumor progression, and the acquisition of CSC traits [61, 62] in solid cancers, including colorectal and breast cancer. Conversely, SETDB1 can oppose TGF-β–induced EMT by repressing SNAI1, as reported in breast epithelial cells [62] and pulmonary fibrosis models [63]. Together, these observations highlight the context-dependent dual role of SETDB1, which can either promote or inhibit EMT. This duality underscores the complexity of the relationships between EMT and CSCs. While early studies proposed a direct and linear association between EMT activation and CSC acquisition [64], more recent data have refined this paradigm. CSCs are now understood as dynamic states shaped by epithelial–mesenchymal plasticity (EMP), with stemness properties confined to specific “stemness windows” often associated with hybrid E/M states [65, 66]. These hybrid states exhibit maximal plasticity, enhanced metastatic potential, and superior tumor-initiating capacity compared to cells locked in purely epithelial or fully mesenchymal states [67, 68]. The positioning of these windows varies across cellular models, ranging from configurations close to the epithelial pole to intermediate hybrid E/M states or more advanced mesenchymal states [65]. In this context, the absence of association between SETDB1 expression and EMT signatures observed in the TCGA HCC cohort suggests that the relevant window for CSC regulation by SETDB1 likely lies closer to the epithelial side.

A major clinical implication of our findings is the strong association between SETDB1 overexpression and resistance to a broad range of HCC therapies, including immunotherapy, TACE, and TKIs. SL tumors display a “hot tumor” immune profile, characterized by higher infiltration of cytotoxic T and NK cells and increased immunogenic signaling, suggesting a greater likelihood of response to immune checkpoint inhibitors (ICIs). In contrast, SH tumors exhibit an “immune-excluded” phenotype with low infiltration of cytotoxic T and NK cells, and enrichment in immunosuppressive pathways. Mechanistically, SETDB1 contributes to this resistance by epigenetically repressing ERVs and interferon‑stimulated genes, thereby dampening type I interferon signaling and reducing tumor immunogenicity [16, 55]. Moreover, SETDB1-high tumors are enriched for gene signatures associated with TACE non-response and sorafenib resistance. These findings align with previous studies [69, 70] and are corroborated by our analysis of the GSE104580 cohort, showing elevated SETDB1 levels in TACE non-responders, thus reinforcing its potential as a resistance biomarker. Importantly, the CSC compartment is a recognized reservoir for therapeutic escape. Our data, together with specific gene signatures, suggest that SETDB1 contributes to this phenotype by modulating drug transporters and anti‑apoptotic pathways [27, 28], thereby sustaining survival under therapeutic pressure. Furthermore, SH tumors are specifically enriched in ferroptosis‑resistant gene programs, suggesting that SETDB1 may suppress ferroptosis-related pathways, a key mechanism of therapeutic failure in HCC.

Taken together, our data showing the strong correlation between SETDB1 expression, stemness features, and tumor aggressiveness highlight SETDB1 as a compelling therapeutic target in HCC. Our chromatin-modifier–based risk score (SETDB1, DNMT3A, TRIM28, HDAC1) shows robust prognostic performance and may help stratify patients for future epigenetic-based interventions. However, selective SETDB1 inhibitors are still in early development. In the absence of direct SETDB1 inhibitors, currently available therapies that modulate SETDB1 indirectly become particularly relevant.

In this context, our experiments with paclitaxel provide important, albeit preliminary, insights. We observed that paclitaxel treatment reduced SETDB1 expression and decreased stemness markers. However, paclitaxel does not directly inhibit SETDB1. Instead, it represses SETDB1 through a p53-dependent mechanism, whereby activated p53 binds the SETDB1 promoter and recruits SUV39H1 to increase H3K9me3 deposition, leading to transcriptional repression [37]. Furthermore, SETDB1 can directly methylate p53 at lysine 370, stabilizing oncogenic mutant p53 proteins by preventing their MDM2-mediated degradation [44]. This p53-mediated repression of SETDB1 is particularly meaningful, as SETDB1 itself transcriptionally silences tumor-suppressor genes, including p53, through H3K9 methylation at specific promoter sites [71], establishing a self-reinforcing oncogenic feedback loop. By reducing SETDB1 levels, paclitaxel disrupts this oncogenic circuitry, allowing reactivation of p53-mediated apoptotic pathways while simultaneously destabilizing gain-of-function p53 mutants that depend on SETDB1-driven methylation for their stability. This dual effect provides a plausible mechanistic basis for the reduced CSC-associated traits observed following paclitaxel exposure.

The reduction in CSC features observed following paclitaxel exposure may therefore reflect broader p53-driven transcriptional reprogramming rather than direct SETDB1 inhibition. As we did not perform SETDB1 knockdown or rescue assays, these findings should be interpreted as correlational. Future mechanistic studies employing genetic SETDB1 inhibition will be essential to establish causality and confirm SETDB1 as a therapeutic vulnerability in HCC.

Future therapeutic approaches may benefit from combination therapies targeting SETDB1 in conjunction with ICIs, ferroptosis inducers, or synthetic lethality partners. Given the functional heterogeneity within SH tumors, precision targeting will require selective inhibitors, refined molecular stratification, and context-specific therapeutic combinations.

In conclusion, our integrative analyses identify SETDB1 as a core epigenetic regulator, supporting CSC identity, immunosuppression, and broad therapy resistance in HCC. SETDB1 defines a clinically aggressive molecular subtype marked by poor differentiation, heightened plasticity, and unfavorable outcomes. These findings highlight SETDB1 as an attractive biomarker and therapeutic target, and they lay the groundwork for future mechanistic studies and translational efforts aimed at incorporating SETDB1-directed strategies into precision medicine for HCC.

## Supplementary Information

Below is the link to the electronic supplementary material.

Supplementary Material 1

Supplementary Material 2

Supplementary Material 3

Supplementary Material 4

Supplementary Material 5

## Data Availability

No datasets were generated or analysed during the current study.

## Abbreviations

AFP: Alpha-fetoprotein
BSA: Bovine Serum Albumin
cDNA: Complementary DNA
CPTAC: Clinical Proteomic Tumor Analysis Consortium
CSCs: Cancer Stem Cells
DAPI: 4’,6-diamidino-2-phenylindole
DFS: Disease-Free Survival
DMEM: Dulbecco’s Modified Eagle’s Medium
FBS: Fetal Bovine Serum
FDR: False Discovery Rate
GDC: Genomic Data Commons
GEO: Gene Expression Omnibus
GPC3: Glypican-3
GSEA: Gene Set Enrichment Analysis
HCC: Hepatocellular Carcinoma
iCCA: Intrahepatic Cholangiocarcinoma
IC50: Half-maximal Inhibitory Concentration
ITH: Intratumoral Heterogeneity
KEGG: Kyoto Encyclopedia of Genes and Genomes
KMTs: Histone Lysine Methyltransferases
LIHC: Liver Hepatocellular Carcinoma
MCC: Matthews Correlation Coefficient
MDR: Multi-Drug Resistance
MSigDB: Molecular Signatures Database
mRNAsi: mRNA-based Stemness Index
NES: Normalized Enrichment Score
PCA: Principal Component Analysis
PBS: Phosphate-Buffered Saline
PPI: Protein-Protein Interactions
PVDF: Polyvinylidene Fluoride
qRT-PCR: Real-time Quantitative Reverse Transcription Polymerase Chain Reaction
RIPA: Radioimmunoprecipitation Assay
ROC: Receiver Operating Characteristic
RSEM: RNA-Seq by Expectation Maximization
SDS-PAGE: Sodium Dodecyl Sulfate Polyacrylamide Gel Electrophoresis
SH: SETDB1 High
SL: SETDB1 Low
ssGSEA: Single Sample Gene Set Enrichment Analysis
TACE: Transarterial Chemoembolization
TCGA: The Cancer Genome Atlas
TIMER: Tumor Immune Estimation Resource
TKI: Tyrosine Kinase Inhibitor
TME: Tumor Microenvironment
TPM: Transcripts Per Million
t-SNE: t-distributed Stochastic Neighbor Embedding
UALCAN: University of Alabama at Birmingham CANcer data analysis Portal
