# Supplementary material for: The epigenetic regulator SETDB1 as a key component of cancer stem cells and drug resistance in primary liver cancer
Source: Cell Oncol (Dordr). 2026 Jan 6;49(1):18. doi: 10.1007/s13402-025-01157-3 (PMC12775003; doi:10.1007/s13402-025-01157-3)
Supplement: Supplementary file 1 — Supplementary Material 1 [file 13402_2025_1157_MOESM1_ESM.pdf]

Fig. 8C

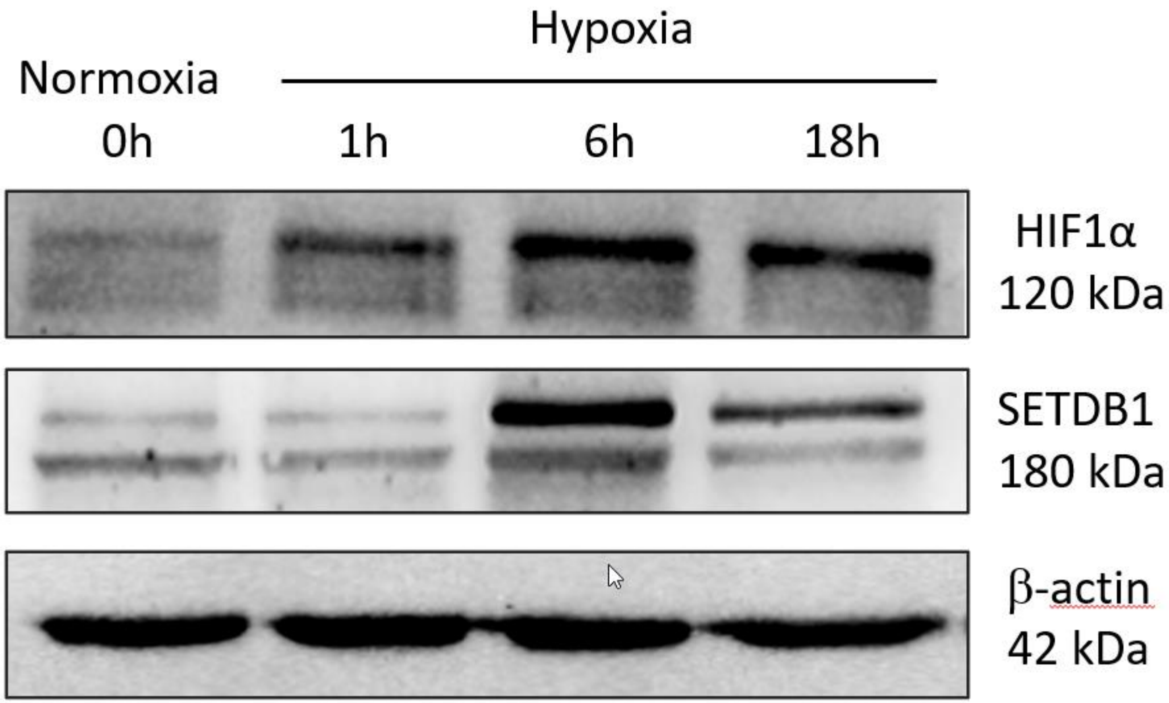

Hypoxia
Normoxia
0h
1h
6h
18h
HIF1α
120 kDa
SETDB1
180 kDa
β-actin
42 kDa

Fig. 8C

Normoxie | **Hypoxie 1h** | **Hypoxie 6h** | **Hypoxie 18h** | Hypoxie 36h

Dépôt protéine totale 35 µg HuH7 (mêmes dépôts que pour SETDB1)

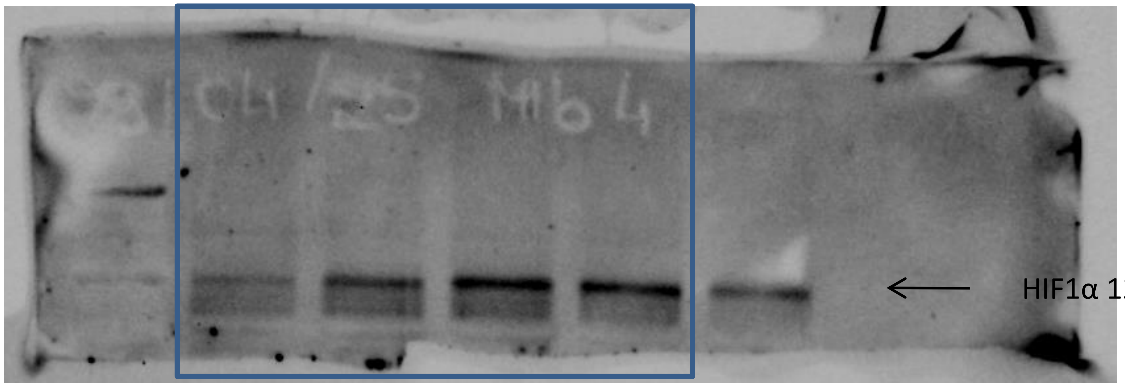

← HIF1α 120 kDa

anti HIF1a BD 610359 (mouse) 1/1000 en TTBS 1X + gelatin 0.2%
anti mouse HRP (Damien) 1/10000 en TTBS 1X + BSA 0.3%

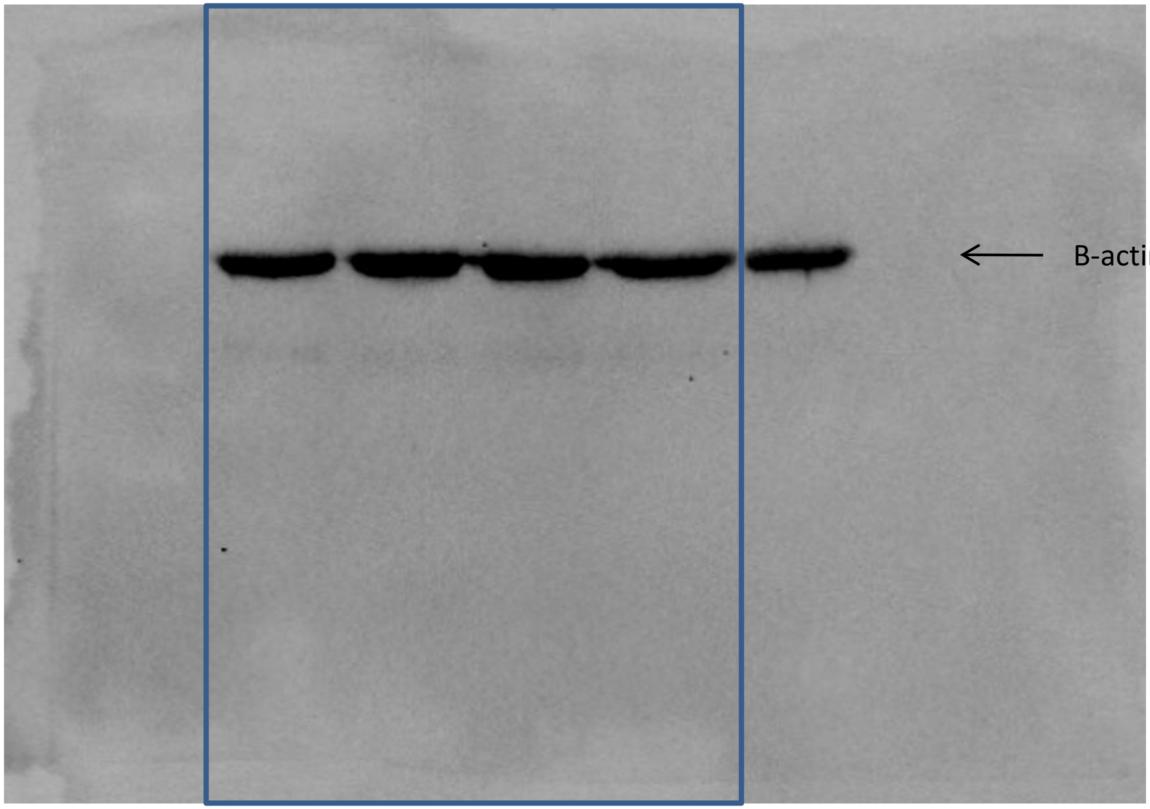

← B-actin 42 kDA

anti b-actin HRP santa-cruz sc-47778 (mouse) 1/10000 en TTBS 1X + gelatin 0.2%

Fig. 8C

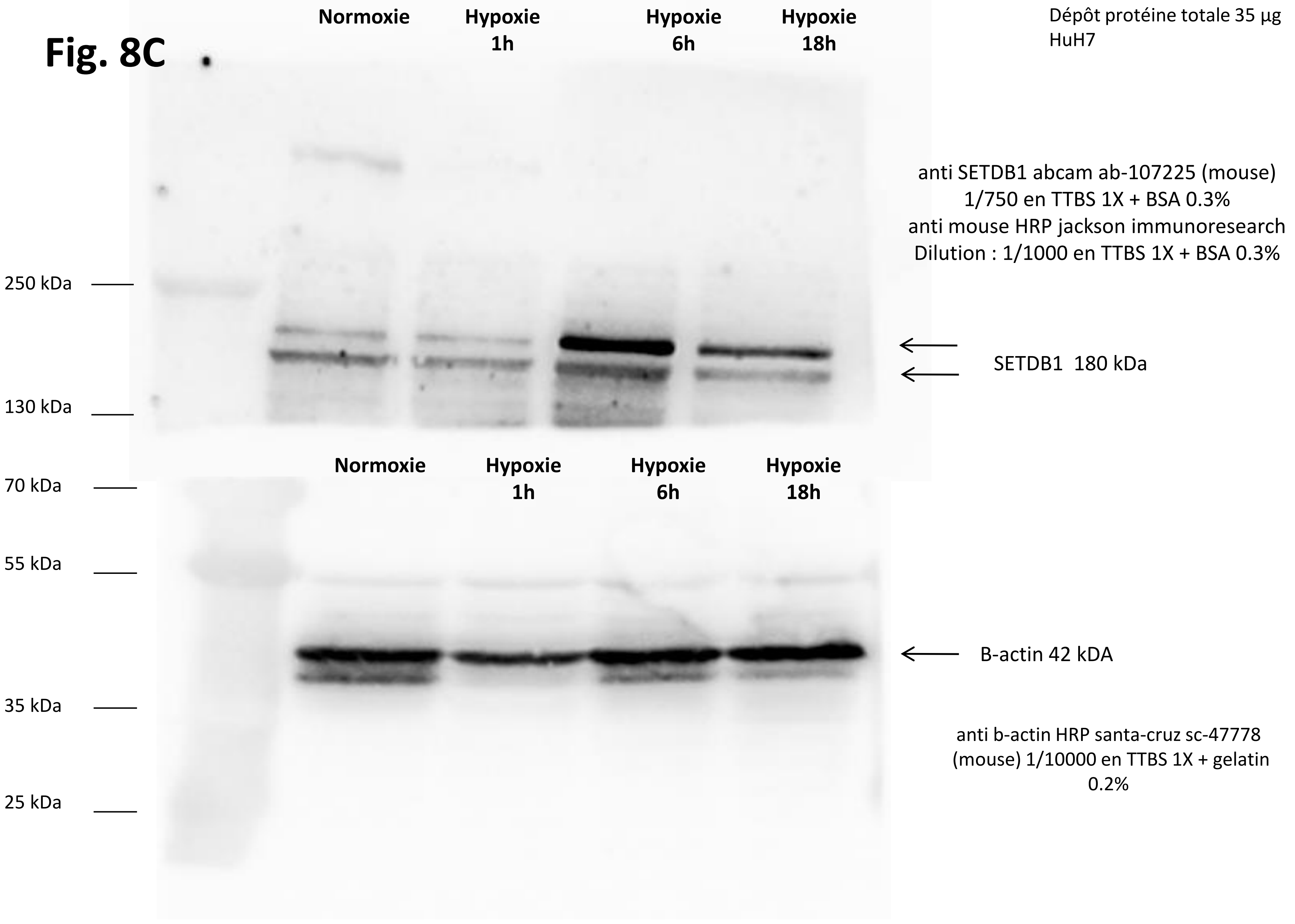
Dépôt protéine totale 35 µg
HuH7
Normoxie
Hypoxie 1h
Hypoxie 6h
Hypoxie 18h
anti SETDB1 abcam ab-107225 (mouse)
1/750 en TTBS 1X + BSA 0.3%
anti mouse HRP jackson immunoresearch
Dilution : 1/1000 en TTBS 1X + BSA 0.3%
250 kDa
130 kDa
SETDB1 180 kDa
70 kDa
55 kDa
35 kDa
25 kDa
Normoxie
Hypoxie 1h
Hypoxie 6h
Hypoxie 18h
B-actin 42 kDA
anti b-actin HRP santa-cruz sc-47778
(mouse) 1/10000 en TTBS 1X + gelatin
0.2%

HuH7WT

Fig. 8H

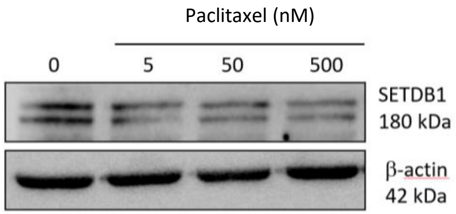

Fig. 8J

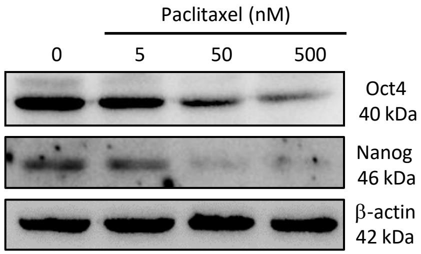

HuH7WT

Fig. 8H

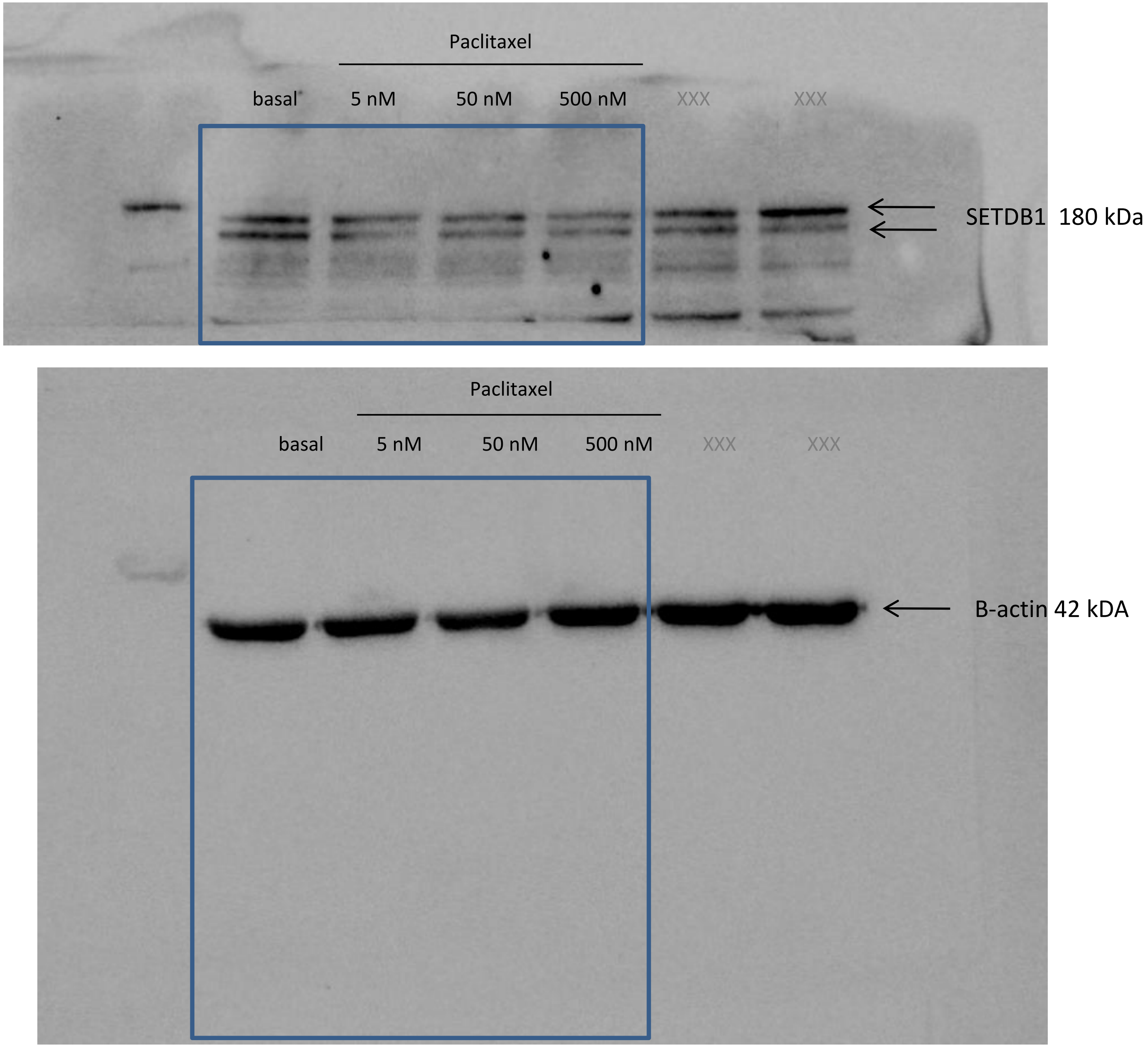

anti SETDB1 abcam ab-107225 (mouse) 1/750 en TTBS 1X + gelatin 0.2%
anti mouse HRP (Damien) 1/10000 en TTBS 1X + BSA 0.3%
anti b-actin HRP santa-cruz sc-47778 (mouse) 1/10000 en TTBS 1X + gelatin 0.2%

HuH7WT

Fig. 8J

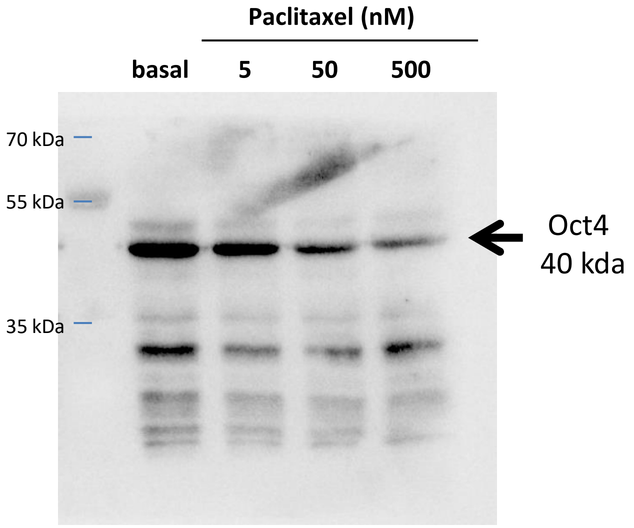

anti Oct4 RetD (rat) 1/100
anti rat HRP 1/1000 en TTBS 1X + BSA 0.3%

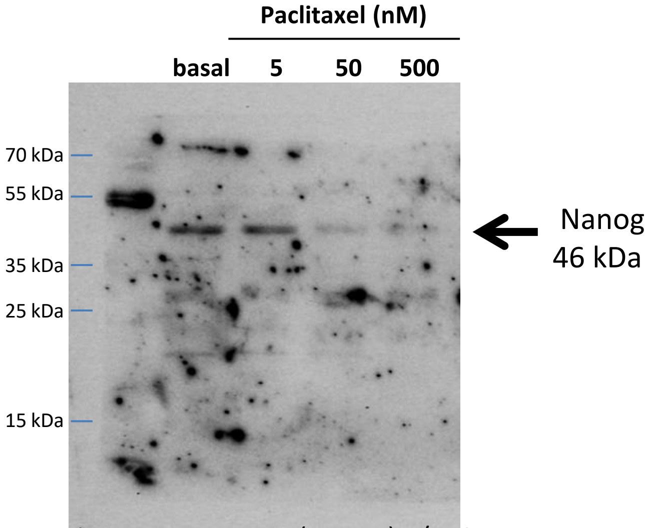

anti nanog santa cruz (mouse) 1/750
anti mouse HRP 1/10000 en TTBS 1X + BSA 0.3%

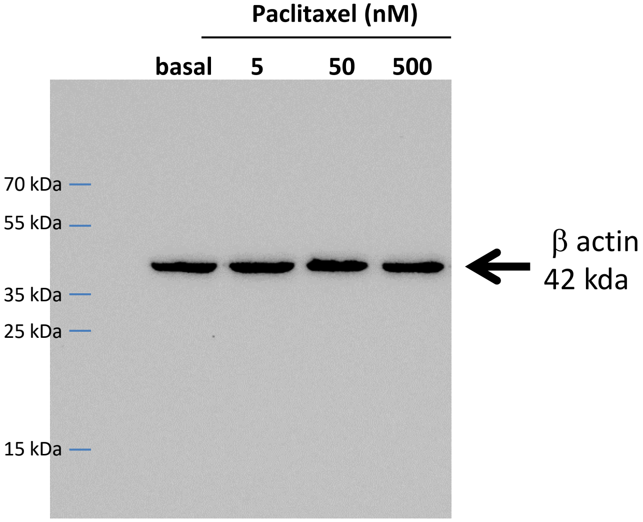

anti b-actin HRP santa-cruz sc-47778
(mouse) 1/10000

HepG2

Suppl Fig. 2

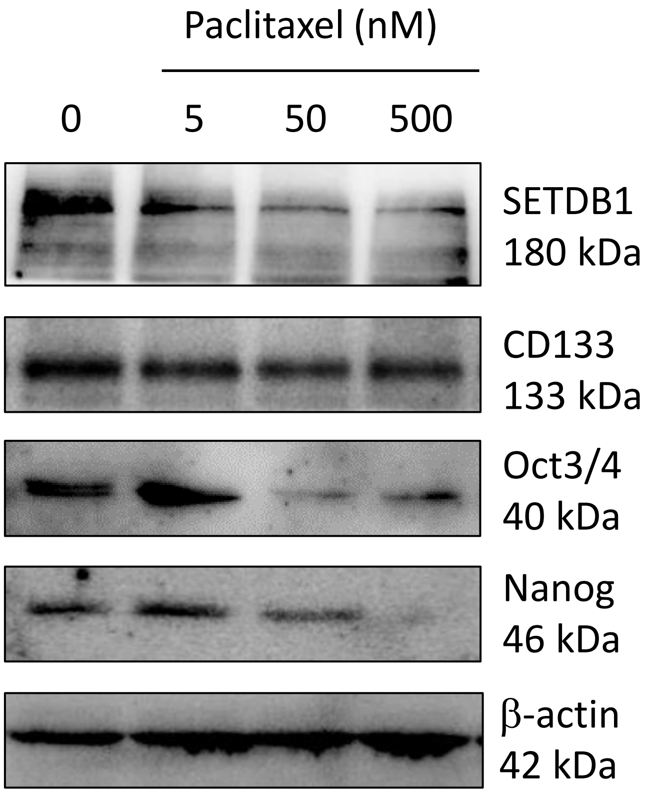

HepG2

Suppl Fig. 2

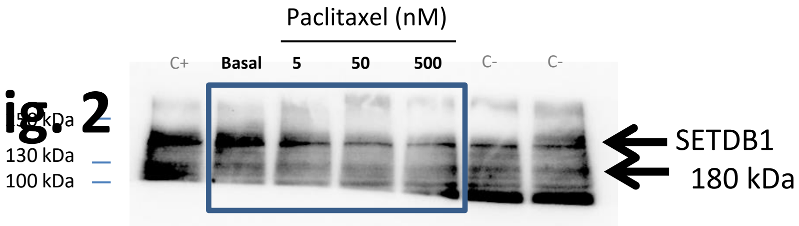

anti SETDB1 abcam (mouse) 1/750
anti mouse HRP 1/10000 en TTBS 1X + BSA 0.3%

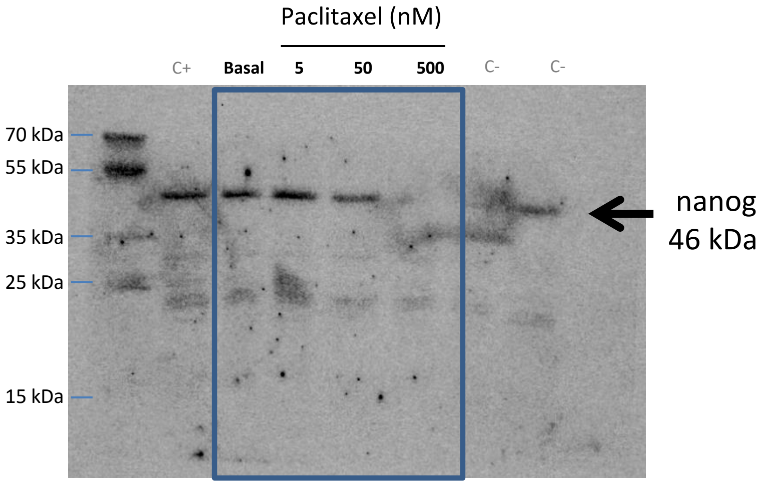

anti nanog santa cruz (mouse) 1/750
anti mouse HRP 1/10000 en TTBS 1X + BSA 0.3%

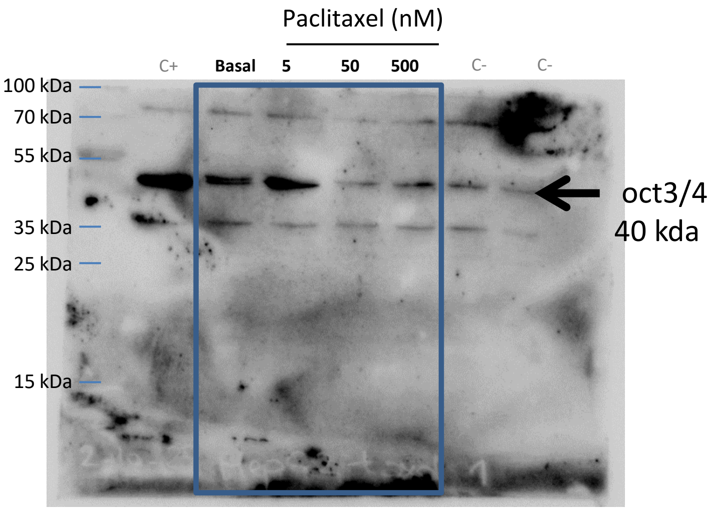

anti oct3/4 RetD (rat) 1/100
anti rat HRP 1/1000 en TTBS 1X + BSA 0.3%

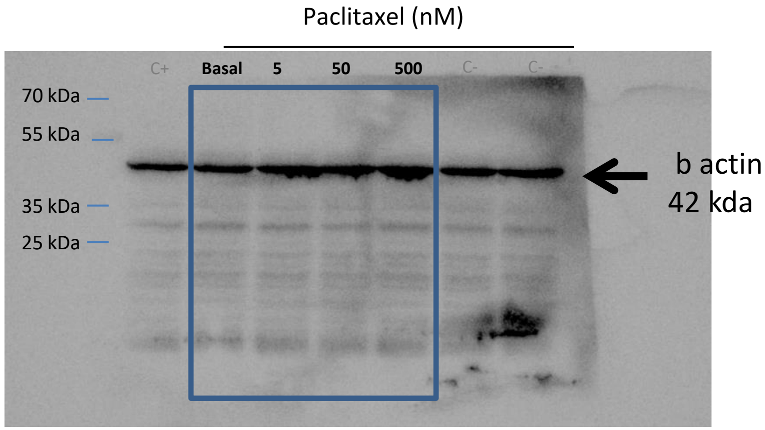

anti b-actin HRP santa-cruz sc-47778
(mouse) 1/10000

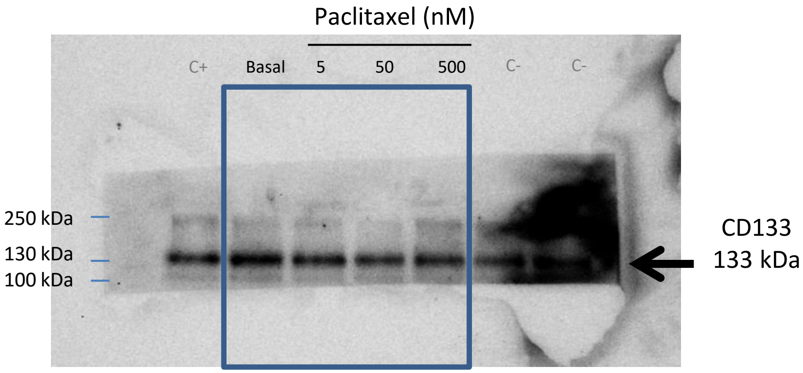

anti CD133 miltenyi(mouse) 1/200
anti mouse HRP 1/10000
