# Supplementary figures and images for: The epigenetic regulator SETDB1 as a key component of cancer stem cells and drug resistance in primary liver cancer
Source: Cell Oncol (Dordr). 2026 Jan 6;49(1):18. doi: 10.1007/s13402-025-01157-3 (PMC12775003; doi:10.1007/s13402-025-01157-3)

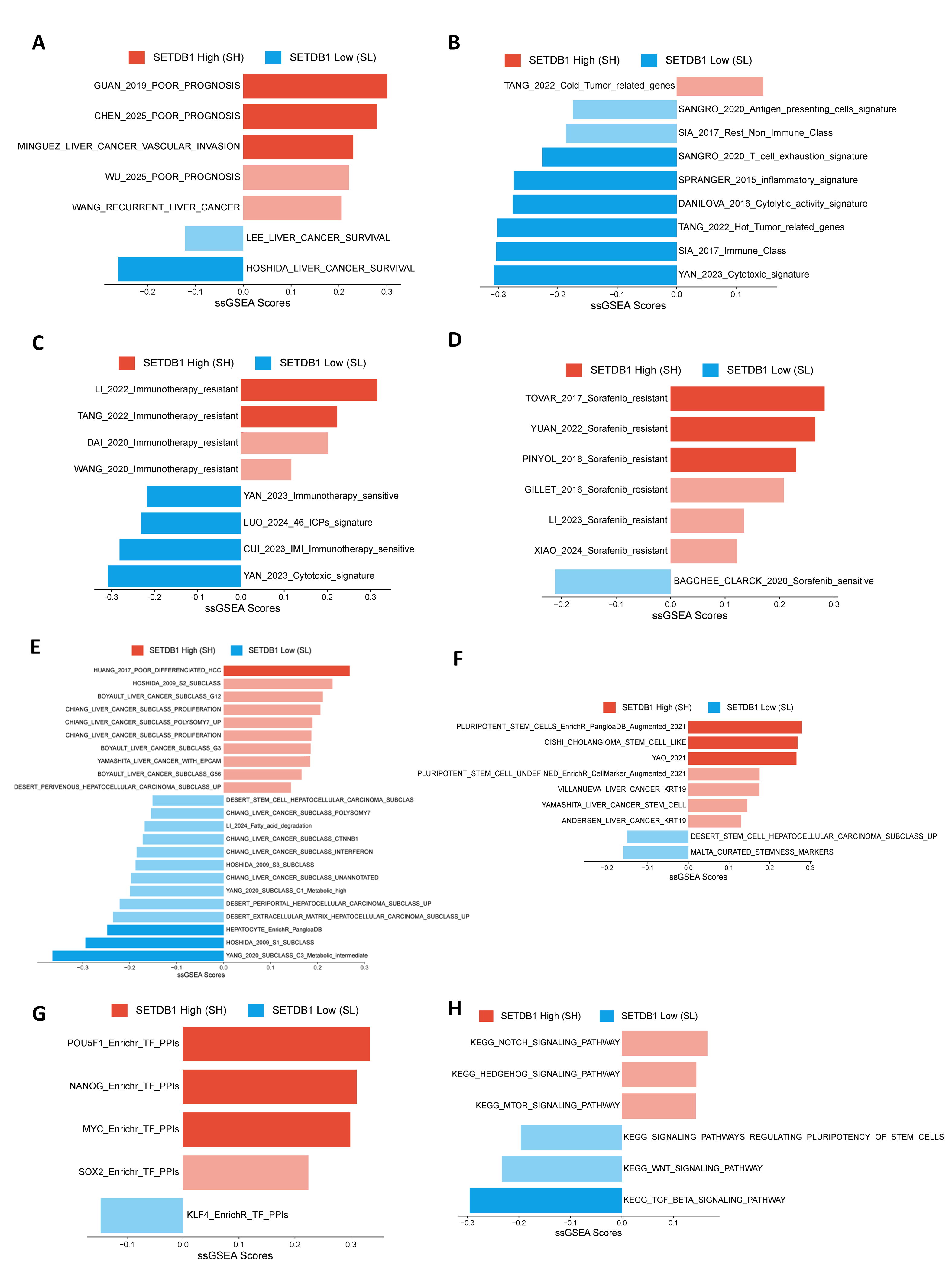

Supplement: Supplementary file 2 — Supplementary Material 2 [file 13402_2025_1157_MOESM2_ESM.tif]

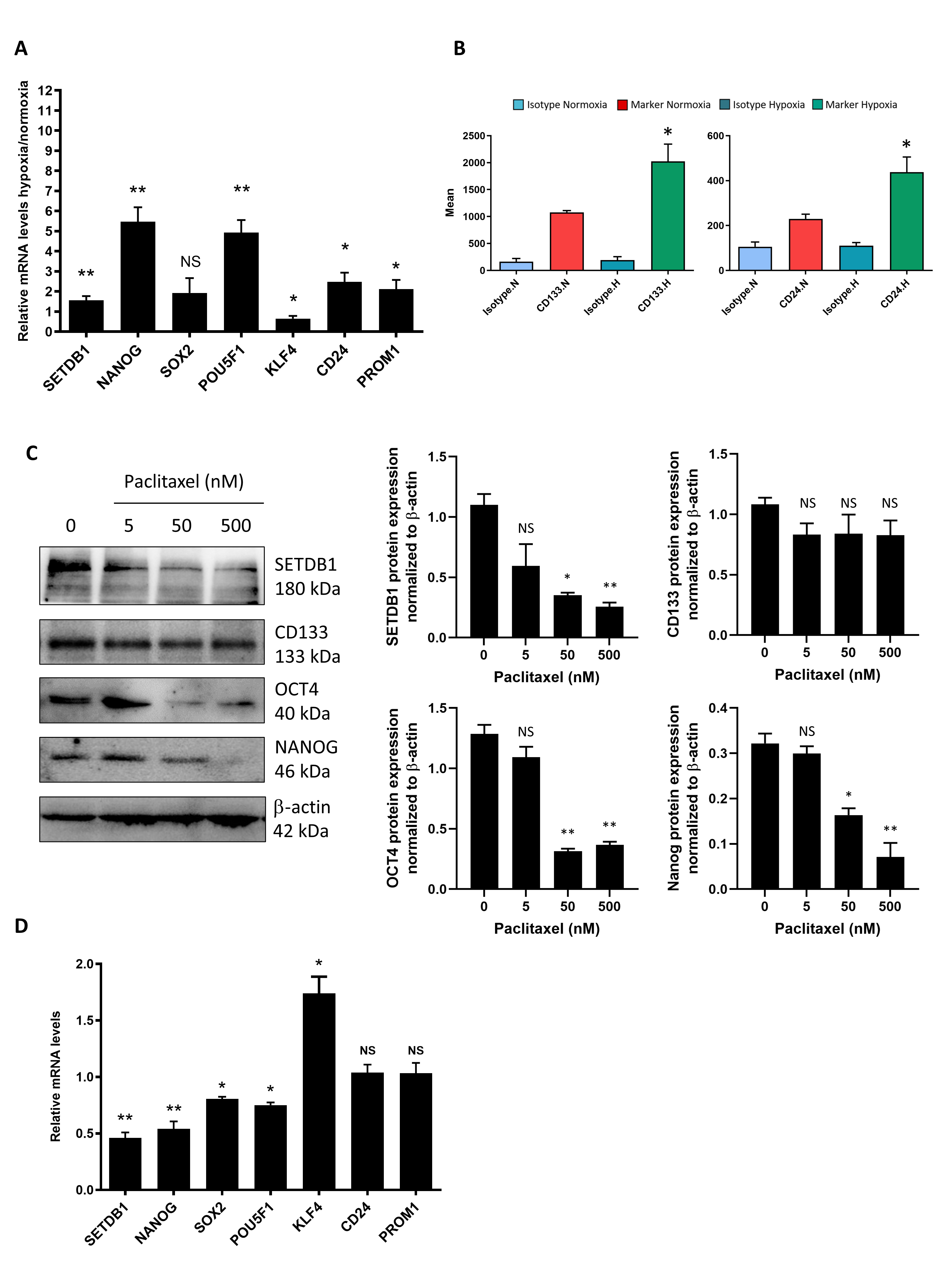

Supplement: Supplementary file 3 — Supplementary Material 3 [file 13402_2025_1157_MOESM3_ESM.tif]
