# Supplementary material for: The epigenetic regulator SETDB1 as a key component of cancer stem cells and drug resistance in primary liver cancer
Source: Cell Oncol (Dordr). 2026 Jan 6;49(1):18. doi: 10.1007/s13402-025-01157-3 (PMC12775003; doi:10.1007/s13402-025-01157-3)
Supplement: Supplementary file 5 — Supplementary Material 5 [file 13402_2025_1157_MOESM5_ESM.docx]

**Supplementary Fig. 1.** **SETDB1-associated transcriptional programs in the GSE104580 cohort.** Bar plot showing ssGSEA enrichment scores for gene signatures associated with poor prognosis **(A)**, immune status **(B)**, immune response **(C)**, sorafenib sensitivity and resistance **(D)**, HCC molecular subclassification **(E)**, and stemness features **(F)** between the SETDB1_High (n = 74) and SETDB1_Low (n = 73) groups. Positive scores (red bars) indicate enrichment in the SETDB1_HIGH group, whereas negative scores (blue bars) indicate enrichment in the SETDB1_LOW group. Gene sets with significant enrichment (P < 0.05, Wilcoxon test) are displayed with saturated colors, while non-significant gene sets (P > 0.05) are shown with attenuated colors.

**Supplementary Fig. 2. SETDB1 expression mirrors stemness dynamics in HepG2 cells under hypoxia and paclitaxel treatment.** **(A)** qPCR analysis of stemness markers in HepG2 cells after 24-hour hypoxia, showing relative mRNA levels of NANOG, SOX2, POU5F1, KLF4, CD24, and PROM1 normalized to 18S and expressed versus normoxia. **(B)** Flow cytometry of stemness-associated surface markers (CD133, CD24, CD44) in HepG2 cells after 3 days of hypoxia versus normoxia. **(C)** Western blot of SETDB1, CD133, OCT4, and NANOG protein expression SETDB1 in HepG2 cells treated with increasing concentrations of paclitaxel (5-500 nM, 48h). The blot and bar graph depict SETDB1 protein levels, with β-actin as loading control. **(D)** qPCR analysis of stemness markers in HepG2 cells treated with paclitaxel (500 nM, 24h), showing relative mRNA levels normalized to 18S and expressed versus control.

**Table S1. Primers and Probes Used for qRT-PCR**. This table details the target genes, their symbols and full names, along with the TaqMan Assay IDs (ThermoFisher) and probe chemistry (FAM-MGB) used for the qRT-PCR analysis.

**Table S2. Baseline characteristics of patients (TCGA-LIHC).** Data are presented as a number and percentage (n(%)). This table shows patient characteristics, including clinical, pathological, and laboratory features, stratified by low versus high SETDB1 expression in LIHC (liver hepatocellular carcinoma) patients.

**Table S3: Gene Signatures Used for GSEA Analysis.** This table lists the gene signatures from various datasets, including signatures associated with hepatic carcinogenesis, tumor subtypes, drug resistance, and immune functions. Each signature is identified by a name, a URL to its source, and a list of the genes that compose it.

**Table S4: Multi-variable disease-free survival model on TCGA liver cancer cohort.** This table presents a multi-variable Cox regression analysis (N=281 with 151 events) for disease-free survival in the TCGA-LIHC cohort. The model includes the SETDB1 expression risk score along with several clinical and pathological variables. The table shows the coefficient beta, hazard ratio, standard error, and P-value for each variable, with a significant P-value (<0.05) for the SETDB1 score.
